# Ultrasound-Assisted Deep Eutectic Solvent-Based Extraction of Polysaccharides from Okra: Optimization by Response Surface Methodology and Artificial Neural Network Modeling

**DOI:** 10.1016/j.ultsonch.2025.107715

**Published:** 2025-12-09

**Authors:** Muhammad Imran, Chih-Huang Weng, Girma Sisay Wolde, Ying-Chen Chen, Yi-Jin Wu, Shang-Ming Huang, Yao-Tung Lin

**Affiliations:** a Department of Soil and Environmental Sciences, National Chung Hsing University, Taichung 40277, Taiwan; b Department of Civil Engineering, I-Shou University, Kaohsiung 84001, Taiwan; c Doctoral Program in Plant Health Care, National Chung Hsing University, Taichung 40277, Taiwan; d Master Program for Food and Drug Safety, College of Medicine, China Medical University, Taichung 40402, Taiwan; e Department of Nutrition, China Medical University, Taichung 40402, Taiwan

**Keywords:** Cavitation, Bioactivity, Antioxidants, Hydrogen bonding, Microstructure

## Abstract

Plant-derived polysaccharides are critical bioactive compounds; however, conventional extraction methods are often inefficient, energy-intensive, and may compromise their bioactivity. Ultrasound-assisted deep eutectic solvent (UA-DES) extraction offers a greener alternative by integrating acoustic cavitation with tunable solvent properties; however, optimization remains complex due to the interaction of multiple processing variables. This study reports a novel application of ultrasound-assisted deep eutectic solvent (UA-DES) extraction for okra polysaccharides (OPs), with process optimization using response surface methodology (RSM) and artificial neural network (ANN) modeling to identify optimal conditions and clarify nonlinear extraction behavior. Among the tested DES systems, choline chloride–citric acid (CCA) exhibited the highest extraction performance. Single-factor experiments and RSM identified sonication time and liquid–solid ratio as key variables. The ANN model achieved higher predictive accuracy than RSM and captured nonlinear and synergistic parameter interactions that were not evident in traditional response surfaces, providing deeper insight into process behavior. Under optimized conditions (2 h, 80 °C, 190 W, 60 mL/g), UA-DES extraction produced 23.56 % OPs and 80.75 % DPPH• scavenging activity, representing 94 % higher yield and 28 % greater antioxidant activity than hot-water ultrasonic (HWU) extraction. UA-DES-derived OPs contained higher contents of uronic acids, total sugars, and glucans, and uniquely included arabinose absent in HWU extracts. Structural analyses revealed pyranose configurations, amorphous crystallinity, and porous microstructures, which collectively contribute to improved solubility and bioactivity. Overall, UA-DES extraction using CCA provides an eco-efficient strategy for producing high-value okra polysaccharides. The integrated RSM–ANN framework enables precise optimization and enhanced mechanistic understanding, supporting UA-DES as a scalable, green technology for the production of functional polysaccharides.

## Graphical abstract

**Figure:**
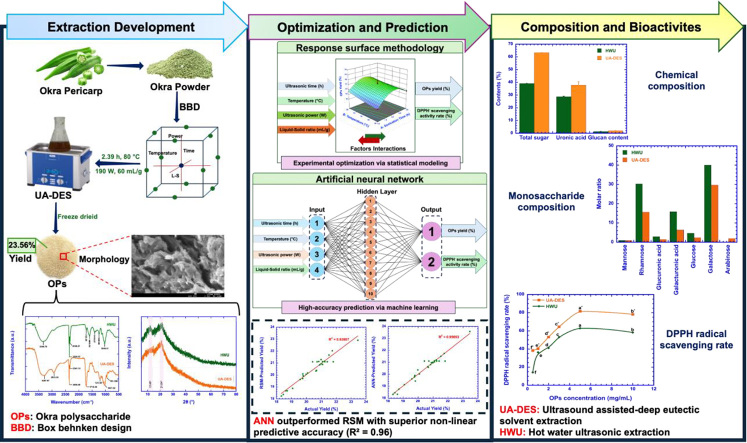

## 1 Introduction

Polysaccharides (PSs) are structurally diverse bio-macromolecules ubiquitously found in plants, algae, fungi, and microorganisms. They possess favorable physicochemical properties such as hydrophilicity, biodegradability, and biocompatibility, which support their wide-ranging applications in the food, nutraceutical, and pharmaceutical industries [1]. Beyond these traditional uses, polysaccharides have also been increasingly applied in biomedical and biotechnological fields, including tissue engineering, wound healing, drug delivery, and cosmetic formulations, as well as in environmental and packaging materials due to their film-forming and moisture-retention capacities [2], [3]. In addition to these physicochemical features, PSs exhibit diverse biological functions, including antioxidant, anti-inflammatory, immunomodulatory, hypoglycemic, and prebiotic activities, which have been systematically documented in recent literature [4], [5]. These multifunctional bioactivities highlight polysaccharides as promising candidates for incorporation into functional foods and therapeutic agents.

Polysaccharides (PSs) exhibit diverse physicochemical and biological properties, making them valuable in various fields, including food, nutraceuticals, pharmaceuticals, and biomedicine. To effectively utilize these bioactive macromolecules, they must first be obtained through appropriate extraction and purification procedures. Extraction refers to the separation of polysaccharides or related components from complex biological matrices using physical, chemical, or enzymatic techniques, enabling their subsequent structural characterization and functional evaluation [6]. The extraction of polysaccharides remains technically challenging due to their hydrophilic nature, structural heterogeneity, and strong interactions with other components in plant matrices [7]. Conventional methods such as hot water ultrasonic extraction (HWU), alkaline extraction (ATE), and enzyme-assisted extraction (ETE) are still widely applied; however, these approaches suffer from critical drawbacks, including long processing times, high energy requirements, polysaccharide degradation, and elevated production costs. Conventional methods, such as hot-water ultrasonic extraction (HWU), alkaline extraction (ATE), and enzyme-assisted extraction (ETE), remain widely applied; however, these techniques exhibit several limitations, including lengthy processing times, high energy consumption, polysaccharide degradation, and increased production costs. In addition, these traditional approaches often yield low extraction efficiencies, commonly below 20 % for many plant matrices, because incomplete cell-wall disruption and thermal or chemical degradation reduce polysaccharide recovery and purity. Recent studies have further shown that such harsh extraction conditions can alter the molecular-weight distribution and decrease biological activity, thereby reducing overall process effectiveness and limiting the functional quality of the extracted polysaccharides [8], [9], [10]. For example, HWU often co-extracts impurities, leading to complicated purification steps, while ATE and ETE can damage polysaccharide structures or result in uneconomical processes. Advanced techniques, including microwave-assisted extraction (MWE), ultrasound-assisted extraction (UAE), and supercritical fluid extraction (SFE), have been developed to address the drawbacks of conventional methods [8]. Recent studies have consistently demonstrated that these techniques offer clear advantages over traditional hot-water extraction. For example, ultrasonic–microwave-assisted extraction has been shown to markedly improve extraction efficiency and antioxidant activity through synergistic cavitation and microwave heating, resulting in enhanced cell-wall disruption and superior product quality [11]. UAE has likewise been reported to increase polysaccharide yield and functional characteristics while reducing both energy consumption and extraction time [12]. Subcritical water extraction provides a solvent-free and environmentally friendly approach that preserves the molecular integrity and bioactivity of polysaccharides [13]. Despite these benefits, challenges such as solvent compatibility, scale-up feasibility, and equipment cost remain, underscoring the continued need for innovative, cost-effective, and sustainable extraction strategies.

In this context, deep eutectic solvents (DESs) have emerged as next-generation green solvents with broad potential for polysaccharide extraction. DESs are typically composed of a hydrogen bond acceptor (HBA), such as choline chloride, betaine, or ethylammonium chloride, combined with a hydrogen bond donor (HBD), such as organic acids, amino acids, or polyols, forming stable eutectic mixtures through hydrogen bonding. Among these, choline chloride is particularly preferred due to its low toxicity, biodegradability, cost-effectiveness, and strong ability to form stable hydrogen-bond networks, which enable efficient extraction of biopolymers under mild conditions. They offer several advantages, including tunable polarity, strong solubilization ability, biodegradability, low toxicity, and recyclability [14]. Importantly, DESs have been successfully applied to extract polysaccharides under mild conditions, preserving both their structural integrity and bioactivity. For instance, natural DES extraction of Paecilomyces hepiali polysaccharides yielded fractions with stronger immunomodulatory activity than those obtained by hot-water extraction, as demonstrated by macrophage activation assays [15]. Similarly, polysaccharides obtained from *Auricularia auricula* using DES displayed superior antioxidant properties compared with those extracted by water or ethanol [16]. Choline chloride-based DESs have also been employed for pectin extraction from olive oil waste (*alperujo*) and fruits such as apple, orange, and strawberry, producing pectins with structural integrity comparable to those obtained by conventional acid-based methods [17]. However, despite their strong solubilization capability, DES-only extractions may still be limited by slow mass transfer and incomplete disruption of plant cell matrices, which can restrict the release of polysaccharides.

To overcome these limitations, recent advances have combined ultrasound-assisted extraction (UAE) with DESs to significantly enhance extraction efficiency and bioactivity. The UAE induces acoustic cavitation, which disrupts cell walls, increases solvent penetration, and accelerates mass transfer. Meanwhile, the hydrogen-bonding network and tunable polarity of DES facilitate the rapid solubilization of target compounds. This complementary mechanism yields a synergistic extraction effect that enhances both yield and functional performance [18], [19]. The ultrasound mechanism involves alternating compression and rarefaction cycles that induce the formation, growth, and collapse of microbubbles within the solvent. The implosion of these cavitation bubbles produces localized high temperature, pressure, and shear stress, leading to the rupture of cell walls, enhanced solvent infiltration, and increased mass transfer. In addition, the microstreaming and turbulence generated by cavitation further improve solute–solvent interactions and diffusion rates, enabling efficient extraction under mild, non-thermal conditions. When combined with the strong hydrogen-bonding capacity and adjustable polarity of DESs, this synergistic system maximizes polysaccharide recovery and maintains bioactivity while reducing energy consumption and solvent toxicity [20], [21], [22]. For example, UAE-DES has been successfully applied to extract polysaccharides from *Ganoderma lucidum*, *Dendrobium officinale*, and *Lycium barbarum*, yielding higher recovery and stronger antioxidant activities compared with conventional solvents [23], [24]. Despite these advances, the application of UAE-DES to okra polysaccharides (OPs) has not been systematically investigated. Okra (*Abelmoschus esculentus* L.) is particularly rich in mucilaginous polysaccharides with documented antioxidant, antidiabetic, and immunomodulatory properties, underscoring its importance as a functional food source [25]. So far, OPs are still predominantly extracted by hot water extraction, resulting in low yield, high energy consumption, and partial loss of bioactivity. To date, no comprehensive study has optimized UAE-DES conditions for OPs recovery, compared their performance with conventional methods, or examined how UAE-DES influences the chemical composition, monosaccharide distribution, and structural characteristics of OPs. Moreover, process optimization is critical for maximizing extraction performance. Response surface methodology (RSM) has been widely employed to analyze parameter interactions and determine optimal conditions, but its quadratic regression model often fails to capture the nonlinear complexity of DES-based systems [26]. Artificial neural networks (ANNs), in contrast, are more effective for modeling nonlinear multivariate relationships and have shown higher predictive accuracy than RSM in polysaccharide extraction studies [27]. Integrating RSM with ANN thus provides a complementary framework, combining the statistical interpretability of RSM with the predictive power of ANN, and offers a robust strategy for optimizing UAE-DES extraction of OPs.

This study aimed to develop and optimize a UA-DES extraction strategy for okra polysaccharides. A series of DESs were screened to identify the most effective solvent system, and extraction parameters including sonication time, temperature, sonication power, and liquid–solid ratio were systematically optimized. This study aimed to develop and optimize a UA-DES extraction strategy for okra polysaccharides. A series of DESs were screened to identify the most effective solvent system, and extraction parameters including sonication time, temperature, sonication power, and liquid–solid ratio were systematically optimized. The performance of the optimized UA-DES method was further compared with that of hot-water ultrasonic (HWU) extraction. Predictive models were established using both RSM and ANN to evaluate parameter interactions and predictive accuracy. The extracted OPs were further characterized for chemical composition, monosaccharide distribution, and structural features by FT-IR, XRD, and SEM. Antioxidant activity was assessed to determine the functional potential of the extracts, and energy consumption was evaluated to highlight the environmental implications of the process. By integrating extraction efficiency, structural characterization, functional evaluation, and sustainability assessment, this study provides a validated framework for producing structurally distinct and bioactive OPs through a green and high-performance approach.

## 2 Materials and methods

### 2.1 Materials and chemicals

Fresh okra was bought from the Farmers Association of Lucao Township (Chiayi County, Taiwan). Choline chloride, citric acid, urea, 1,4-butanediol, glycerol, malic acid, ethylene glycol, D-glucuronic acid, D-galactose, L-rhamnose, D-mannose, D-arabinose, 1-phenyl-3-methyl-5-pyrazolone, and inulin were purchased from Sigma-Aldrich (USA). D-glucose was obtained from Merck Millipore (USA), and additional reagents and solvents were supplied by JT Baker (USA). All chemicals were of analytical grade, and deionized water was used throughout the experiments.

### 2.2 Preparation and characterization of deep eutectic solvents (DESs)

**Fig. 1:**
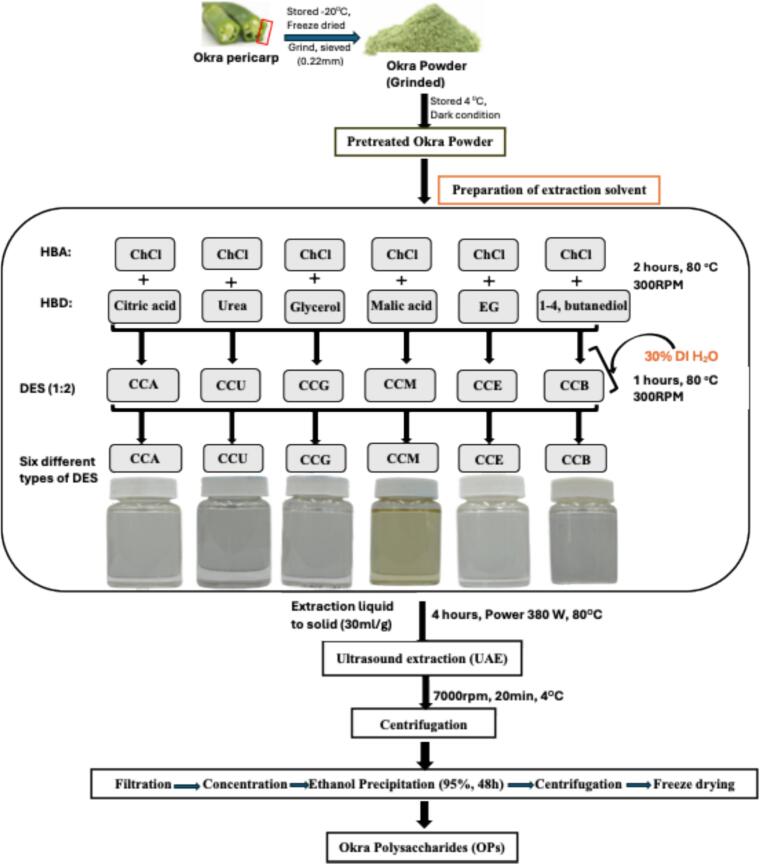
Flow chart illustrating the ultrasound-assisted deep eutectic solvent (UA-DES) extraction of okra polysaccharides (OPs). Fresh okra pericarps were stored at − 20 °C, freeze-dried, ground, and sieved (0.22 mm) to obtain okra powder, which was stored at 4 °C under dark conditions before extraction. Six types of deep eutectic solvents (DESs) were prepared by combining choline chloride (ChCl, hydrogen bond acceptor, HBA) with various hydrogen bond donors (HBDs) at a 1:2 M ratio: citric acid (CCA), urea (CCU), glycerol (CCG), malic acid (CCM), ethylene glycol (CCE), and 1,4-butanediol (CCB). Each DES mixture was heated and stirred at 80 °C and 300 rpm for 2 h, followed by the addition of 30 % (v/v) deionized water and further stirring for 1 h to enhance solvent homogeneity. Pretreated okra powder was mixed with each DES at a liquid-to-solid ratio of 30 mL/g and subjected to ultrasound-assisted extraction (UAE) for 4 h at 80 °C and 380 W sonication power. After extraction, the mixture was centrifuged (7000 rpm, 20 min, 4 °C), and the supernatant was collected for polysaccharide recovery. Extracts were filtered, concentrated, and precipitated with 95 % ethanol for 48 h. The precipitated polysaccharides were collected by centrifugation, washed, and freeze-dried to obtain purified okra polysaccharides (OPs).

DESs were prepared by mixing choline chloride (CC) as the hydrogen bond acceptor (HBA) with citric acid, urea, glycerol, malic acid, ethylene glycol, or 1,4-butanediol as hydrogen bond donors (HBDs) at a 1:2 M ratio. The mixtures were heated at 80 °C for 3 h until homogeneous liquids were obtained. To reduce viscosity, 30 % (w/w) deionized water was added to each DES (Fig. 1). Six CC-based DESs were thus generated and designated as CCA (choline chloride-citric acid), CCU (choline chloride-urea), CCG (choline chloride-glycerol), CCM (choline chloride-malic acid), CCE (choline chloride-ethylene glycol), and CCB (choline chloride-1,4-butanediol), respectively. The viscosity, pH, and conductivity of each DES formulation were measured before extraction to evaluate solvent properties, and the results are provided in the Supplementary Data (Table S1).

### 2.3 Extraction of okra polysaccharides

Fresh okra was washed, seeds were removed, and the material was freeze-dried. The dried samples were ground into a fine powder. For preliminary solvent screening, 1 g of okra powder was mixed with 30 mL of each DES and subjected to sonication in a thermostatic ultrasonic bath (Elma sonic P 300H, Germany) at 80 °C for 4 h, with a power of 380 W and frequency of 37 kHz (Fig. 1). OPs extraction efficiency was evaluated based on yield (%) and 2,2-diphenyl-1-picrylhydrazyl (DPPH^•^) radical scavenging activity (%). For comparison, hot water ultrasonic (HWU) and hot water bath (HWB) extractions were also performed using deionized water as the solvent. HWU was conducted under the same ultrasonic conditions as the DES extractions, whereas HWB was carried out at 80 °C for 4 h without ultrasound. After extraction, the mixtures were centrifuged at 7000 rpm for 20 min at 4 °C using a refrigerated centrifuge (ROTANTA 460R, Andreas Hettich GmbH & Co. KG, Tuttlingen, Germany). The supernatants were filtered through qualitative filter paper (Advantech, 55 mm diameter) and collected using a vacuum pump (EYELA A-1000S, China). The filtrates were then precipitated with four volumes of 95 % ethanol at 4 °C for 48 h, and the resulting precipitates were freeze-dried to obtain crude OPs. The extraction yield of OPs (%) was calculated as follows:

(1) $$Yield\left(\%\right)=\frac{\mathrm{Weight}\;\mathrm{of}\;\mathrm{OPs}}{\mathrm{Weight}\;\mathrm{of}\;\mathrm{powder}}\times 100$$

### 2.4 Single-factor experiment for okra polysaccharides extraction

Single-factor experiments were conducted to evaluate the effects of four independent variables on OPs yield: liquid-to-solid ratio (30, 40, 50, and 60 mL/g), sonication time (1, 2, 3, and 4 h), extraction temperature (50, 60, 70, and 80 °C), and sonication power (114, 190, 266, and 342 W). Each parameter was varied individually while the others were kept constant, and all experiments were performed in triplicate.

### 2.5 Optimization and validation of okra polysaccharides extraction by RSM

A Box–Behnken design (BBD) in response surface methodology (RSM) was applied to evaluate the effects of sonication time (A), extraction temperature (B), sonication power (C), and liquid-to-solid ratio (D) on OPs yield and DPPH^•^ scavenging activity. Experimental conditions were selected based on preliminary studies (Table S2), and each variable was coded at three levels (–1, 0, +1). RSM was implemented using Design-Expert software (version 8.0.7.1, Minneapolis, USA). A total of 29 randomized experimental runs, including factorial and center points, were conducted, and the corresponding parameters and responses are summarized in Table 1.

**Table 1:** Experimental runs with observed and predicted values of extraction yield and DPPH radical scavenging activity for ultrasound-assisted DES extraction of OPs using RSM and ANN models.

Run	Independent variables				OPs yield (%)					DPPH^•^ scavenging activity (%)				
						RSM		ANN			RSM		ANN	
	A	B	C	D	Actual	Predicted	Residual	Predicted	Residual	Actual	Predicted	Residual	Predicted	Residual
1	2	70	266	40	20.98	20.94	0.04	20.96	0.02	78.34	77.61	0.73	77.97	0.37
2	2	80	190	60	23.45	22.91	0.54	23.58	−0.13	81.00	79.75	1.25	80.90	0.10
3	2	70	190	50	21.80	21.11	0.69	21.11	0.69	79.11	77.34	1.76	77.39	1.72
4	2	70	114	60	21.09	21.12	−0.03	21.13	−0.04	77.73	78.00	−0.27	77.90	−0.17
5	1	70	190	60	19.81	19.91	−0.10	19.44	0.37	75.08	75.44	−0.35	75.32	−0.24
6	3	80	190	50	20.09	20.17	−0.08	20.03	0.06	75.86	75.51	0.35	75.94	−0.08
7	2	60	190	60	21.09	20.89	0.20	21.04	0.05	77.78	77.37	0.41	78.12	−0.34
8	2	80	190	40	20.89	20.85	0.04	20.98	−0.09	76.30	76.65	−0.35	76.66	−0.36
9	1	70	114	50	18.62	18.68	−0.06	18.33	0.29	73.18	73.12	0.06	73.15	0.03
10	2	60	190	40	20.83	21.13	−0.30	20.81	0.02	75.34	76.53	−1.18	75.57	−0.23
11	2	70	190	50	20.42	21.11	−0.69	21.11	−0.69	75.74	77.34	−1.60	77.39	−1.65
12	2	80	114	50	20.98	21.29	−0.31	21.01	−0.03	76.84	77.48	−0.64	77.58	−0.74
13	2	70	266	60	21.74	22.03	−0.29	21.75	−0.01	78.54	78.68	−0.14	78.15	0.39
14	3	70	114	50	19.08	18.88	0.20	19.13	−0.05	73.92	73.71	0.21	74.20	−0.28
15	1	70	190	40	18.32	18.24	0.08	18.32	0.00	72.59	72.22	0.37	72.64	−0.05
16	1	60	190	50	18.45	18.37	0.08	18.46	−0.01	72.84	72.73	0.11	72.58	0.26
17	3	70	190	60	19.76	20.09	−0.33	19.79	−0.03	74.83	75.72	−0.89	75.22	−0.39
18	2	80	266	50	21.51	21.67	−0.16	21.50	0.01	77.58	78.15	−0.57	77.72	−0.14
19	2	60	266	50	21.21	21.15	0.06	21.21	0.00	77.92	77.81	0.12	77.72	0.21
20	1	70	266	50	18.72	18.68	0.04	18.76	−0.04	73.62	73.77	−0.14	73.45	0.17
21	1	80	190	50	19.58	19.63	−0.05	19.62	−0.04	74.56	74.60	−0.04	74.49	0.07
22	3	70	190	40	19.78	19.93	−0.15	19.91	−0.13	74.83	74.99	−0.17	74.98	−0.15
23	2	70	190	50	20.71	21.11	−0.40	21.11	−0.40	76.06	77.34	−1.28	77.39	−1.33
24	2	70	114	40	20.68	20.39	0.29	20.59	0.09	75.71	75.12	0.59	75.42	0.29
25	3	60	190	50	19.75	19.70	0.05	19.78	−0.03	75.37	74.87	0.49	76.13	−0.76
26	2	70	190	50	21.24	21.11	0.13	21.11	0.13	77.73	77.34	0.39	77.39	0.34
27	2	70	190	50	21.40	21.11	0.29	21.11	0.29	78.07	77.34	0.73	77.39	0.68
28	3	70	266	50	20.64	20.34	0.30	20.64	0.00	76.25	76.24	0.01	76.21	0.03
29	2	60	114	50	19.98	20.07	−0.09	20.37	−0.39	75.37	75.32	0.05	75.53	−0.16

A: Sonication time (h), B: Extraction temperature (°C), C: Sonication power (W), D: L-S ratio (mL/g).

The extraction yield and antioxidant activity were modeled using a second-order polynomial Eq. (2):

(2) $$Y=\beta_{0}+\sum_{i=1}^{4}\beta_{i}X_{i}+\sum_{i=1}^{4}\beta_{\mathrm{ii}}X_{i}^{2}+\sum_{i=1}^{4}\sum_{j=i+1}^{4}\beta_{\mathrm{ij}}X_{i}X_{j}$$

where Y is the predicted response, β_0_ is the intercept, β_i_ are the linear coefficients, β_ii_ are the quadratic coefficients, β_ij_ are the interaction coefficients, and X_i_ and X_j_ are the coded levels of the independent variables.

Model adequacy was evaluated using regression statistics, including R^2^, adjusted R^2^, adequate precision (AP), prediction error sum of squares (PRESS), and coefficient of variation (CV). Response surface and contour plots were used to visualize factor interactions and to identify optimal extraction conditions. The accuracy of the model was further confirmed by comparing predicted and experimental results for OPs yield and DPPH^•^ scavenging activity.

### 2.6 Modeling and prediction of okra polysaccharides extraction by ANN

The MATLAB neural network fitting tool was used to establish an ANN model for OPs extraction (Fig. S1). The ANN consisted of four input variables (X_1_–X_4_), one hidden layer, and an output layer with two response variables: OPs yield and DPPH^•^ scavenging activity. The model was trained iteratively to minimize the error between experimental and predicted values, using the Levenberg–Marquardt backpropagation algorithm (trainlm), which is widely recognized for its speed and accuracy in nonlinear function approximation. Weights and biases were optimized as adjustable model parameters. The dataset was randomly divided into 70 % for training, 15 % for validation, and 15 % for testing. A hyperbolic tangent sigmoid (tansig) transfer function was used in the hidden layer, while a linear (purelin) function was employed in the output layer.

### 2.7 Performance evaluation of RSM and ANN models

The predictive performance of RSM and ANN was evaluated using statistical indices: coefficient of determination (R^2^), mean squared error (MSE), root mean squared error (RMSE), mean absolute percentage error (MAPE), and mean absolute deviation (MAD, reported as a supplementary index rather than a primary criterion [28]. The equations used are:

(3) $$\mathrm{MSE}=\frac{1}{n}\sum_{i=1}^{n}{\left(Y-X\right)}^{2}$$

(4) $$\mathrm{RMSE}=\sqrt{\frac{1}{n}\sum_{i=1}^{n}{\left(Y-X\right)}^{2}}$$

(5) $$\mathrm{MAD}=\frac{1}{n}\sum_{i=1}^{n}\left(Y-X\right)$$

(6) $$\mathrm{MAPE}=\frac{100}{n}\sum_{i=1}^{n}\left|\frac{Y-X}{Y}\right|$$

(7) $$R^{2}=1-\frac{\sum_{i=1}^{n}{\left(Y-X\right)}^{2}}{\sum_{i=1}^{n}{\left(Y-Z\right)}^{2}}$$

where *n* is the number of observations, *Y* is the actual yield, *X* is the predicted yield, and *Z* is the mean of the actual OPs yield and DPPH^•^ scavenging activity. The ANN learning process was monitored by tracking the evolution of MSE across training epochs. Training was automatically stopped when the validation error increased for six consecutive iterations, thereby minimizing overfitting and ensuring generalization performance.

### 2.8 Physicochemical characterization of okra polysaccharides

Fourier transform infrared spectroscopy (FT-IR) spectra of OPs were obtained using an FT-IR-4700 spectrometer (JASCO, Japan). Measurements were performed in the 4000–400 cm^−1^ range using attenuated total reflection (ATR) mode at a resolution of 4 cm^−1^ with 128 scans. Samples (≈2 mg) were ground with spectroscopic-grade KBr and pressed into pellets to improve signal quality. The major absorption bands were assigned to hydroxyl stretching, C–H vibrations, carboxyl groups, and glycosidic linkages, allowing identification of characteristic polysaccharide functional groups**.**

Surface morphology of OPs was examined with a JSM-7800F scanning electron microscope (SEM) (JEOL, Japan). Samples were mounted on aluminum stubs using double-sided conductive carbon tape and sputter-coated with a thin layer of gold (≈10 nm) under vacuum to minimize charging artifacts. Imaging was conducted at an accelerating voltage of 3 kV at magnifications ranging from 500 × to 30,000 × . SEM micrographs were evaluated for particle size, surface roughness, porosity, and evidence of cell wall disruption.

X-ray diffraction (XRD) patterns of OPs were obtained using a PANalytical X’Pert Pro MRD diffractometer (Malvern PANalytical, USA). Samples were scanned over a 2θ range of 5–80° at a scanning rate of 2°/min, with Cu Kα radiation (λ = 1.5406 Å) operating at 40 kV and 30 mA. The relative crystallinity index was calculated by integrating the area under crystalline and amorphous peaks, providing a quantitative assessment of OPs structural order. The broad peaks were interpreted as evidence of amorphous domains typically observed in polysaccharides.

### 2.9 Antioxidant activity of okra polysaccharides

The DPPH radical scavenging activity of OPs was determined using a modified method [29]. OPs solutions were prepared at different concentrations ranging from 0.50 to 10 mg/mL. A 2 mL aliquot of each sample solution was mixed with 2 mL of freshly prepared DPPH^•^ solution (0.1 mM in ethanol) in triplicate. The mixtures were shaken thoroughly and incubated in the dark at 25 °C for 30 min. Absorbance was recorded at 517 nm using a UV–Vis spectrophotometer. A DPPH^•^ solution without the sample was used as the blank. Ascorbic acid (0.1–1.0 mg/mL) was used as a positive control for comparison, and all measurements were performed in triplicate to ensure reproducibility. The scavenging activity (%) was calculated as:

(8) $$Scavengingactivity\left(\%\right)=\frac{A_{\mathrm{blank}}-A_{\mathrm{sample}}}{A_{\mathrm{blank}}}\times 10$$

where A_blank_ is the absorbance of the control (DPPH solution without sample) and A_sample_ is the absorbance of the test solution.

### 2.10 Determination of okra polysaccharides chemical composition

#### 2.10.1 Total sugar and glucan contents

Total sugar content was determined by the phenol–sulfuric acid method. A 5 % phenol solution was prepared in deionized water, and glucose solutions (0–400 µg/mL) were prepared as standards from a 1 mg/mL stock. For the assay, the sample or standard solution was mixed with 5 % phenol and concentrated sulfuric acid at a 1:1:5 ratio. The mixture was vortexed, incubated for 30 min, and the absorbance was recorded at 490 nm. Total sugar content was quantified using the glucose calibration curve. The β-glucan content was determined following the method of McCleary and Draga [30] with a β-glucan assay kit (K-YBGL, Megazyme, Ireland).

#### 2.10.2 Uronic acid content

The uronic acid content was determined using the Blumenkrantz and Asboe-Hansen method [31]. Briefly, a 200 μL OPs sample was placed in a microcentrifuge tube (Eppendorf), to which 1.2 mL of 0.0125 M sodium tetraborate/sulfuric acid (H_2_SO_4_) was added. The resulting mixture was cooled in an ice bath for 5 min, then heated in a dry bath at 100°C for another 5 min and subsequently returned to the ice bath for an additional 5 min. Following this, 20 μL of m-hydroxydiphenyl (3-Phenylphenol) was introduced to initiate the color development reaction. After a 15-minute incubation period, 200 μL of the reaction mixture was transferred to a 96-well plate, where the absorbance was measured at 520 nm using an Infinite 200 ELISA reader. A standard curve was established using glucuronic acid standards at varying concentrations (0, 12.5, 25, 50, 100, and 200 mg/L) for quantification.

#### 2.10.3 Monosaccharide composition

Monosaccharide composition was determined by PMP derivatization followed by HPLC, based on a previously reported method with minor modifications [32]. Approximately 10 mg of polysaccharide samples was hydrolyzed in 8 mL of 2 M trifluoroacetic acid (TFA) at 110 °C for 6 h. Hydrolysates (200 µL) and monosaccharide standards were mixed with 240 µL of 0.3 M NaOH and 240 µL of 0.5 M 1-phenyl-3-methyl-5-pyrazolone (PMP) in methanol, vortexed, and incubated at 70 °C for 2 h. After neutralization with 240 µL of 0.3 M HCl, the samples were extracted three times with chloroform, and the aqueous phase was filtered through a 0.22 µm membrane. HPLC analysis was performed on a HITACHI system equipped with a UV detector set at 245 nm. The mobile phase consisted of 0.05 M phosphate buffer and acetonitrile (84:16, v/v), with a flow rate of 1.0 mL/min, column temperature at 40 °C, and injection volume of 20 µL. The monosaccharide standards included D-(+)-mannose, L-(+)-rhamnose, D-(+)-glucuronic acid, D-(+)-galacturonic acid, D-(+)-glucose, D-(+)-galactose, and D-(−)-arabinose.

### 2.11 Statistical analysis

All experimental data were analyzed using SPSS Statistics version 22.0 (IBM Corp., Armonk, NY, USA). Results are expressed as mean ± standard deviation (SD), and error bars in figures indicate SD. Each experiment was performed in triplicate unless otherwise stated. Differences between groups were assessed by one-way analysis of variance (ANOVA) followed by Tukey’s post hoc test. Prior to ANOVA, data were checked for normal distribution (Shapiro–Wilk test) and homogeneity of variances (Levene’s test) to ensure validity of parametric analysis. Statistical significance was considered at *P* < 0.05.

## 3 Results and discussion

### 3.1 Single-factor experimental analysis

**Fig. 2:**
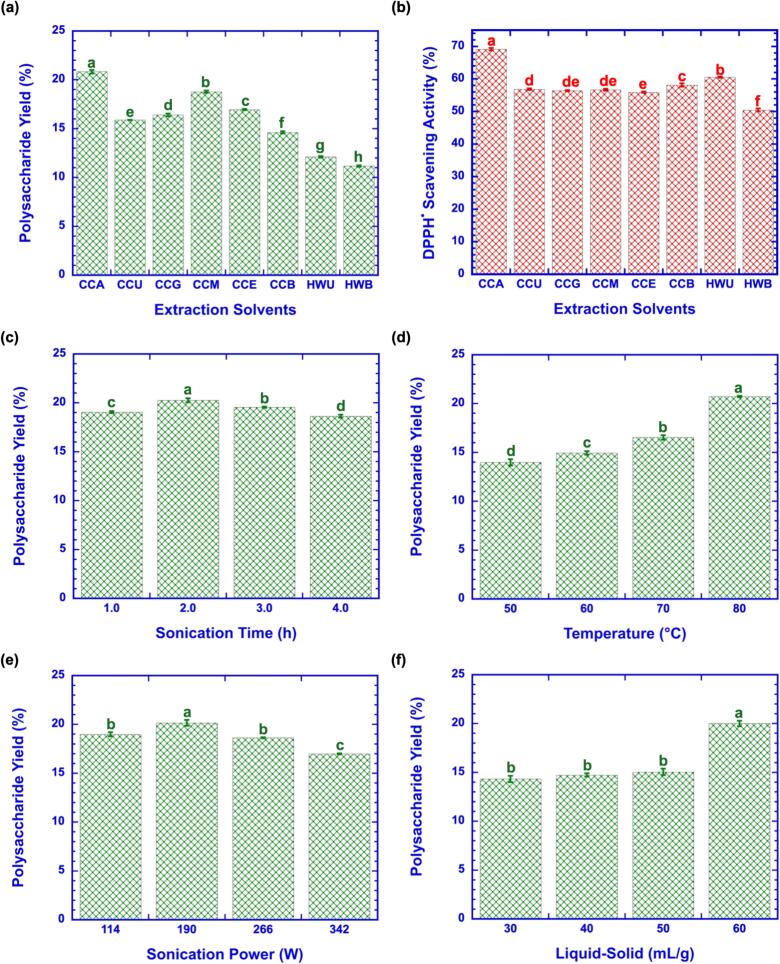
Effects of extraction solvents and ultrasound-assisted extraction parameters on the yield and antioxidant activity of okra polysaccharides. (a) Polysaccharide yield with different extraction solvents. (b) DPPH radical scavenging activity with different extraction solvents. (c) Effect of sonication time on polysaccharide yield. (d) Effect of extraction temperature on polysaccharide yield. (e) Effect of sonication power on polysaccharide yield. (f) Effect of liquid-to-solid ratio on polysaccharide yield. Data are expressed as mean ± SD (n = 3). Different letters above the bars indicate significant differences among groups, as determined by one-way ANOVA followed by Tukey’s post hoc test. Statistical significance was considered at *p* < 0.05. OPs Experimental conditions: OPs concentration of 5 mg/mL, DPPH^•^ concentration was 0.1 mM.

The physicochemical properties of extraction solvents strongly influence polysaccharide recovery, as diffusion, solubility, viscosity, and polarity determine solvent penetration and mass transfer efficiency [33]. As shown in Fig. 2a, the yield of OPs varied markedly among DESs. Among the tested solvents, CCA combined with ultrasound-assisted extraction (UA) achieved the highest yield (20.82 %), demonstrating its superior solubilization efficiency. CCM (18.77 %) also showed relatively high efficiency. Notably, citric acid–based DESs exhibited exceptional extraction performance, likely due to their strong acidity (pH 2.53–4.42), which promotes disruption of the plant matrix and enhances polysaccharide solubilization. In contrast, sugar-based DESs, which maintain near-neutral pH, and alcohol-based DESs, which are relatively basic, demonstrated poor performance, suggesting that solvent acidity is a key determinant of OPs recovery. This trend is consistent with previous studies reporting that acidic DESs facilitate dissolution of acidic polysaccharides by weakening hydrogen bonding and chelating metal ions within the cell wall [34].

The antioxidant activity of OPs obtained with different DESs is shown in Fig. 2b. Consistent with yield, CCA-OPs exhibited the highest DPPH^•^ scavenging activity (69.44 %), followed by CCB (58.04 %), whereas CCE had the lowest activity (56.15 %). Both HWB (50.67 %) and HWU (60.54 %) showed lower scavenging activity than CCA. The superior activity of CCA-derived OPs may be attributed not only to higher yield but also to selective solubilization of uronic acid–rich fractions and favorable modification of monosaccharide composition. Previous work demonstrated that acidic DES extraction enhances the recovery of bioactive sugar residues and improves antioxidant activity compared with conventional solvents [35].

In the single-factor experiments (Fig. 2c–f), sonication time, temperature, power, and liquid–solid ratio each influenced OPs yield. Yield peaked at 2 h (20.27 %) and decreased at 4 h (18.65 %), suggesting polysaccharide hydrolysis during prolonged heating. Increasing temperature enhanced yield up to 80 °C (20.72 %), while lower recovery at 50 °C (13.97 %) was likely due to insufficient thermal energy to disrupt the plant matrix. This indicates that a synergistic balance between ultrasonic energy and moderate thermal input is required: sufficient to disrupt the cell wall, but not excessive to degrade polysaccharides [28]. Sonication power influenced extraction efficiency, with the yield increasing from 114 W to 190 W (20.15 %) but decreasing at 342 W (16.99 %). Moderate cavitation at 190 W effectively disrupted okra cell walls and promoted solvent penetration, but excessive cavitation at 342 W generated shear forces capable of breaking glycosidic bonds, reducing recovery [36]. This agrees with reports on other polysaccharides, where over-intensified ultrasound damaged structural integrity and decreased extraction efficiency. The liquid–solid ratio (L–S) also played an important role (Fig. 2f). Maximum yield was obtained at 60 mL/g (20.01 %), while lower ratios limited solvent penetration (30 mL/g, 14.32 %). Although increasing L–S enhances extraction by improving mass transfer and solubilization, ratios above 60 mL/g were not tested, as further increases may be economically impractical for large-scale production. Collectively, these single-factor results indicate that acidic DESs, particularly CCA, provide a favorable environment for OPs extraction and bioactivity enhancement. The findings highlight the dual role of solvent chemistry (pH, hydrogen bonding capacity) and extraction conditions (time, temperature, power, L–S) in determining efficiency. While preliminary optimal were identified (2 h, 80 °C, 190 W, 60 mL/g), multi-factor optimization via RSM and ANN was essential to confirm these trends and account for parameter interactions. Table S3 provides a comparative assessment of previously published polysaccharide extraction methods from various plant sources, emphasizing the effectiveness and relevance of the current extraction methodology.

In addition, the comparative analysis with HWU and literature data (Table S3) further demonstrates the superiority of the UA-DES system. UA-DES achieved a yield of 23.56 % polysaccharide within 120 min, while consuming 0.38 kWh. In contrast, HWU required 240 min and 1.52 kWh to reach only 12.11 %. This represents approximately 50–75 % reductions in both extraction time and energy expenditure. UA-DES, therefore, combines higher productivity with lower operational cost. The enhanced performance results from the joint effects of acoustic cavitation and the hydrogen-bonding network of DES, which together promote rapid solvent diffusion, efficient cell wall disruption, and better solubilization of polysaccharides. These outcomes confirm that the UA-DES method developed in this study for extracting plant polysaccharides from okra provides an energy-efficient, high-yield, and functionally superior alternative to conventional extraction and other assisted extraction techniques.

### 3.2 Box-Behnken design (BBD) and response surface analysis

The effects of four independent variables (sonication time X_1_, extraction temperature X_2_, sonication power X_3_, and liquid–solid ratio X_4_) on OPs yield and DPPH^•^ scavenging activity were evaluated using a Box–Behnken design (BBD). A total of 29 experimental runs with factorial and center points (Table 1) provided the design, observed responses, and predicted values. The predicted and experimental values of both OPs yield, and antioxidant activity showed close concordance. The extraction yield ranged from 18.32 % to 23.45 %, while DPPH^•^ scavenging activity varied between 72.59 % and 81.0 %. This relatively narrow range, particularly at higher levels, suggests that UA-DES extraction is inherently efficient, but subtle optimization of process parameters further enhances performance.

#### 3.2.1 Optimizing parameters using response surface methodology

The correlation between responses and factors was modeled by quadratic regression, yielding the following second-order polynomial Eqs. (9), (10):

(9) $$Y\;\left(\%\right)=4.91+0.0810A+0.0707B+0.0054C+0.0444D-0.0402AB+0.0228AC-0.0162AD-0.0208BC+0.0563BD+0.0257CD-\;0.{1937A}^{2}-{0.0100B}^{2}-{0.0246C}^{2}+{0.0034D}^{2}$$

(10) $$Y\;\left(\%\right)=8.79+0.0443A+0.0358B+0.0453C+0.0562D-0.0180AB+0.0271AC-0.0362AD-0.0260BC+0.0314BD-0.0260CD-\;0.{1690A}^{2}+{0.0016B}^{2}-{0.0105C}^{2}+{0.0111D}^{2}$$

where Y represents OPs yield (Eq. (9) or DPPH^•^ scavenging activity (Eq. (10), and A–D denote coded levels of sonication time, temperature, sonication power, and liquid–solid ratio, respectively.

ANOVA results (Table 2) indicated that both models were highly significant, with *P*-values less than 0.0001 and 0.0004 and high *F*-values (16.80 and 6.96) for yield and DPPH^•^ activity, respectively. The correlation coefficients (R^2^ = 0.944 for yield, 0.874 for DPPH^•^ activity) demonstrated excellent agreement between predicted and experimental values. Adjusted R^2^ values (0.888 and 0.749) and predicted R^2^ values (0.809 and 0.571) further confirmed robustness, while low coefficients of variation (CV) supported precision. Lack-of-fit tests yielded p-values of 0.938 and 0.936, indicating no significant lack of fit.

**Table 2:** Analysis of variance (ANOVA) for the response surface quadratic model of extraction yield and DPPH radical scavenging activity from ultrasound-assisted DES extraction of OPs.

	OPs yield (%)						DPPH^•^ scavenging rate (%)					
Source	Sum of squares	df	Mean square	F-value	P-value		Sum of squares	df	Mean square	F-value	P-value	
Model	0.4305	14	0.0308	16.800	< 0.0001	****	0.3240	14	0.0231	6.9600	0.0004	***
A-Sonication time	0.0338	1	0.0338	18.480	0.0007	***	0.0236	1	0.0236	7.1000	0.0185	*
B-Temperature	0.0268	1	0.0268	14.650	0.0018	**	0.0154	1	0.0154	4.6200	0.0495	*
C-Sonication power	0.0194	1	0.0194	10.610	0.0057	**	0.0246	1	0.0246	7.4100	0.0165	*
D-Liquid-Solid	0.0296	1	0.0296	16.160	0.0013	**	0.0380	1	0.0380	11.420	0.0045	**
AB	0.0021	1	0.0021	1.1400	0.3031		0.0013	1	0.0013	0.3913	0.5417	
AC	0.0067	1	0.0067	3.6500	0.0768		0.0029	1	0.0029	0.8853	0.3627	
AD	0.0075	1	0.0075	4.0800	0.0629		0.0053	1	0.0053	1.5800	0.2292	
BC	0.0015	1	0.0015	0.8317	0.3772		0.0027	1	0.0027	0.8143	0.3821	
BD	0.0148	1	0.0148	8.1000	0.0129		0.0039	1	0.0039	1.1900	0.2942	
CD	0.0003	1	0.0003	0.1907	0.6690		0.0027	1	0.0027	0.8130	0.3825	
A^2^	0.2526	1	0.2526	137.96	< 0.0001	****	0.1852	1	0.1852	55.740	< 0.0001	****
B^2^	0.0012	1	0.0012	0.6436	0.4358		0.0000	1	0.0000	0.0073	0.9331	
C^2^	0.0030	1	0.0030	1.6400	0.2206		0.0007	1	0.0007	0.2145	0.6503	
D^2^	0.0031	1	0.0031	1.6800	0.2157		0.0008	1	0.0008	0.2395	0.6322	
Residual	0.0256	14	0.0018				0.0465	14	0.0033			
Lack of fit	0.0112	10	0.0011	0.3128	0.9381	ns	0.0206	10	0.0021	0.3177	0.9356	ns
Pure error	0.0144	4.0	0.0036				0.0259	4.0	0.0065			
Cor total	0.4561	28					0.3705	28				
R^2^ = 0.944; R^2^_adj_ = 0.888; R^2^_Pred_ = 0.809; C.V. (%) = 0.947; Adeq Precision = 16.72							R^2^ = 0.874; R^2^_adj_ = 0.749; R^2^_Pred_ = 0.571; C.V. (%) = 0.661; Adeq Precision = 10.40					

Note: ns = non-significant, df = degree of freedom.

For OPs yield, the most influential factor was the quadratic term of sonication time (A^2^), followed by liquid–solid ratio (D), sonication time (A), temperature (B), sonication power (C), and the BD interaction, in the order A^2^ > D > A > B > C > BD. For DPPH^•^ scavenging activity, the order was A^2^ > D > C > A > B. These findings highlight sonication time and solvent loading (L–S) as the dominant parameters controlling OPs recovery and bioactivity, which is consistent with previous reports on ultrasound-assisted polysaccharide extraction [37].

#### 3.2.2 Analysis of response surface methodology

**Fig. 3:**
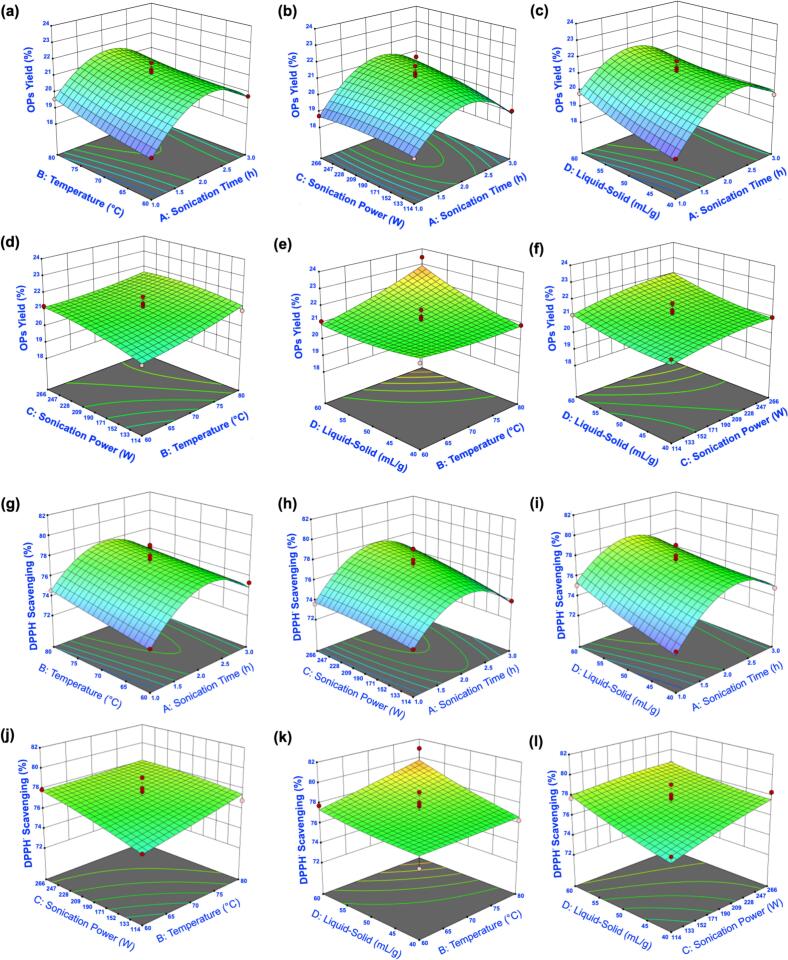
Response surface plots showing the interactive effects of extraction parameters on polysaccharide yield and DPPH radical scavenging activity of okra polysaccharides. (a–f) Interaction effects of extraction temperature, sonication time, sonication power, and liquid-to-solid ratio on OPS yield. (g–l) Interaction effects of the same parameters on DPPH radical scavenging activity.

Three-dimensional response surface and contour plots (Fig. 3, Fig. S2) visualized parameter effects and interactions. As shown in Fig. 3a–c, OPs yield increased with sonication time but decreased with prolonged exposure, consistent with degradation under extended treatment. Fig. 3d shows a similar pattern for sonication power: yield increased up to 190 W but decreased at 342 W, reflecting a balance between cavitation-induced cell wall disruption and excessive shear that damages polysaccharides [37]. Liquid–solid ratio exerted a strong positive influence on yield (Fig. 3e), with no decline observed within the tested range. Fig. 3f revealed that yield was highest when both L–S and sonication power increased, but excessive power negated the benefit of higher solvent volume. For DPPH^•^ scavenging activity, the response surfaces showed analogous trends. Fig. 3g–i indicated that antioxidant activity increased with sonication time before declining at prolonged durations. Fig. 3, Fig. 3 confirmed that 190 W was optimal for activity, while higher powers caused a decline. Fig. 3l showed that combining higher sonication power with a high L–S ratio improved scavenging activity, but activity diminished at excessive power. These patterns reinforce that extraction efficiency and antioxidant activity are both governed by an optimal energy window: sufficient to release polysaccharides, but not so intense as to degrade them.

#### 3.2.3 Verification of predictive model

The optimized conditions predicted by RSM were 2 h sonication time, 80 °C, 190 W power, and 60 mL/g L–S, yielding 23.56 % OPs. Under similar conditions, maximum DPPH^•^ scavenging activity was 80.75 % at 2 h sonication. The experimentally obtained yield (23.56 %) closely matched the predicted value (23.47 %), and the antioxidant activity (80.75 %) was also consistent with the prediction (79.76 %), validating the model. Compared with HWB (yield 11.16 %, scavenging 50.67 %) and HWU (yield 12.11 %, scavenging 60.81 %), the optimized UA-DES method nearly doubled yield and markedly enhanced antioxidant activity. This confirms that the RSM model accurately captured process trends and demonstrated clear superiority of UA-DES over conventional extraction methods.

### 3.3 ANN modeling and predictive evaluation

**Fig. 4:**
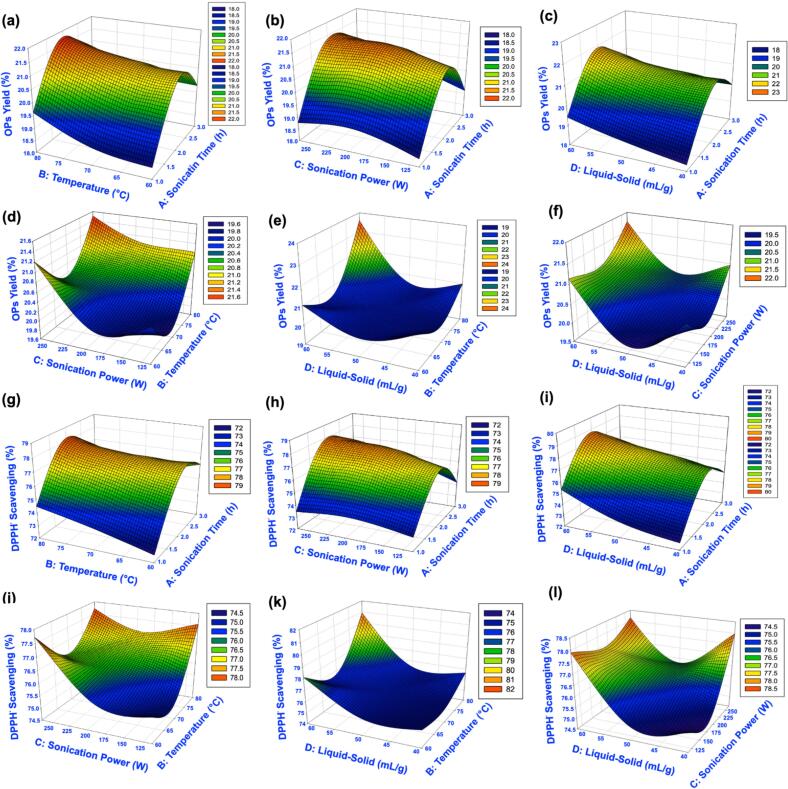
Response surface plots predicted by the ANN model showing the interactive effects of extraction parameters on polysaccharide yield and DPPH radical scavenging activity of okra polysaccharides. (a–f) Interaction effects of extraction temperature, sonication time, sonication power, and liquid-to-solid ratio on OPS yield. (g–l) Interaction effects of the same parameters on DPPH radical scavenging activity.

The RSM experimental dataset with four inputs (sonication time, temperature, sonication power, and liquid–solid ratio) and two outputs (OPs yield and DPPH^•^ scavenging activity) was used to train and test the ANN model in a single network. The three-dimensional response surface plots (Fig. 4) illustrate the effects of input variables on OPs yield and antioxidant activity predicted by ANN. For OPs yield (Fig. 4a–f), the model predicted increases with temperature and sonication time, although yield declined under prolonged heating. Optimal sonication power and time improved recovery, while excessive power decreased it. Yield also increased with the liquid–solid ratio until reaching a maximum, after which further increases produced no benefit and even slight reductions. Synergistic effects between sonication power and temperature, and between liquid–solid ratio and temperature, were evident at moderate conditions. The highest yield was obtained at approximately 190 W sonication power and the maximum liquid–solid ratio tested.

For DPPH^•^ scavenging activity (Fig. 4g–l), optimal values were obtained when temperature, power, liquid–solid ratio, and time were balanced. Deviations above these optima reduced antioxidant activity, reinforcing the significance of controlled energy input. This consistency between yield and activity highlights that the same extraction conditions promote both efficiency and functionality. Contour plots (Fig. S3) confirmed that the optimum region was approximately 2.0–2.5 h sonication time, 75–80 °C, 150–300 W power, and 60 mL/g solvent ratio.

#### 3.3.1 Model accuracy and error analysis

**Fig. 5:**
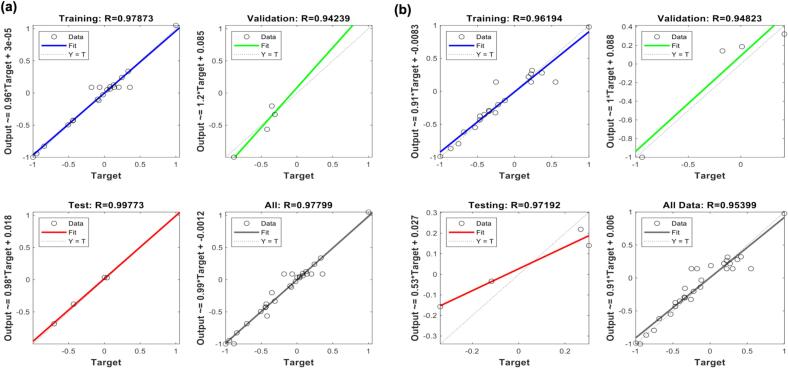
Evaluation of the TRAINLM (Levenberg–Marquardt algorithm) ANN model based on regression plots for training, validation, testing, and overall datasets in predicting extraction yield and DPPH radical scavenging activity of okra polysaccharides. (a–d) Regression plots of OPs yield in the training, validation, testing, and overall datasets. (e–h) Regression plots of DPPH radical scavenging activity in the training, validation, testing, and overall datasets.

The ANN model trained with RSM experimental data demonstrated strong predictive capability for OPs yield and DPPH^•^ scavenging activity. As shown in Table 3, the ANN achieved MSE of 0.014 and RMSE of 0.24 for OPs yield, and 0.0118 and 0.60 for antioxidant activity. The model also yielded MAD of 0.14, MAPE of 0.69 %, and R^2^ of 0.96 for yield, and 0.40, 0.53 %, and 0.91 for activity, confirming its accuracy. Correlation coefficients across datasets were 0.978, 0.997, 0.942, and 0.977 for yield, and 0.962, 0.972, 0.948, and 0.954 for antioxidant activity, indicating excellent generalization (Fig. 5). Regression plots demonstrated close alignment between experimental and predicted values. Learning curves (Fig. S4a–d) showed that both training and validation errors decreased until reaching an optimal point, after which validation error slightly increased, and training was stopped after six consecutive errors to prevent overfitting. Gradient values of 0.0267 for yield and 1.488 × 10^−12^ for antioxidant activity confirmed convergence stability. Error histograms (Fig. S4e and f) showed most residuals clustered near zero with minimal skewness, supporting robustness. The ANN model predicted maximum OPs yield of 23.58 % under optimum conditions (2 h, 80 °C, 190 W, 60 mL/g), and maximum antioxidant activity of 80.90 %. These values closely matched experimental measurements (23.56 % yield and 80.75 % scavenging activity), confirming accuracy of the model. Compared with RSM, ANN provided finer resolution in identifying the optimum region and yielded predictions more closely aligned with experimental values, particularly for DPPH^•^ activity where RSM showed larger deviations. Maximum antioxidant activity was 80.90 % under similar conditions. These predictions closely matched experimental values, confirming accuracy. Compared with RSM, ANN provided finer resolution of the optimum region and more precise predictive capability. Direct comparison further emphasized ANN’s advantages. ANN achieved higher R2 (0.96 vs. 0.94), lower RMSE (0.24 vs. 0.28), lower MAD (0.14 vs. 0.21), and lower MAPE (0.69 % vs. 1.0 %) than RSM. Both models were statistically robust according to ANOVA, but ANN better captured non-linear relationships between process parameters and responses. Although RSM demonstrated higher adequacy precision (17.61), which confirms its reliability for process optimization, ANN outperformed RSM in predictive accuracy due to its inherent ability to handle non-linear, multivariate interactions [38]. This suggests that RSM and ANN should be viewed as complementary, with RSM providing mechanistic interpretation and statistical significance of factor effects, and ANN enhancing predictive precision for complex systems [39].

**Table 3:** Comparison of statistical parameters for RSM and ANN models in predicting extraction yield and DPPH radical scavenging activity of OPs.

		OPs yield	DPPH^•^ scavenging activity rate
RMSE	RSM	0.28	0.71
	ANN	0.24	0.60
MAD	RSM	0.21	0.53
	ANN	0.14	0.40
MAPE (%)	RSM	1.00	0.69
	ANN	0.69	0.53
R^2^	RSM	0.94	0.87
	ANN	0.96	0.91

#### 3.3.2 Validation of model predictions

**Fig. 6:**
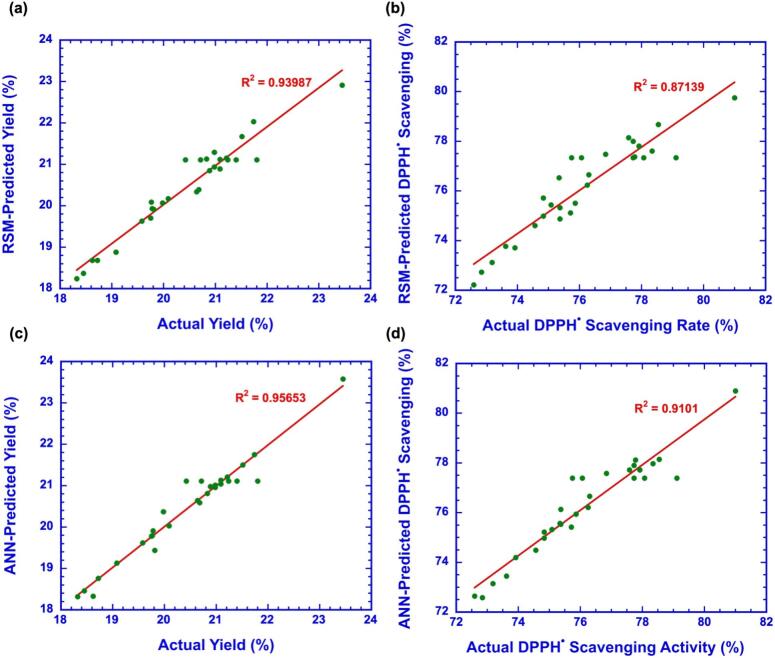
Comparison of RSM and ANN models for predicting extraction yield and DPPH radical scavenging activity of okra polysaccharides. (a–b) Correlation between actual and predicted values of yield and DPPH^•^ activity using the RSM model. (c–d) Correlation between actual and predicted values of yield and DPPH^•^ activity using the ANN model.

Both training and test datasets achieved R2 values above 0.94, confirming the ANN model’s strong fit and generalization capability (Fig. 6). ANN-predicted values showed closer alignment with experimental data compared with RSM, as indicated in Table S4. While RSM successfully captured overall extraction trends (Fig. 6, Fig. 6), ANN predictions were more tightly clustered around the experimental results (Fig. 6, Fig. 6). Residual analysis further supported model adequacy, the normal probability plots of externally studentized residuals showed a nearly linear distribution along the reference line (Fig. S5), indicating that errors were normally distributed without systematic bias. The ANN model outperformed the RSM model by providing higher predictive accuracy and revealing nonlinear parameter interactions that were not visible in the RSM plots. The RSM surfaces (Fig. 3 d–f and Fig. 3 j–l) showed nearly linear responses with minimal interaction effects, whereas the ANN surfaces (Fig. 4 d–f and Fig. 4 j–l) exhibited distinct nonlinear and synergistic relationships between sonication power and extraction temperature on yield, and between sonication time and liquid-to-solid ratio on antioxidant activity. This demonstrates that the ANN model not only improved prediction performance but also offered a valuable advantage by capturing the actual nonlinear behavior of the extraction system. These results demonstrate that ANN not only provided superior predictive accuracy but also validated the robustness of the RSM model by confirming that RSM-predicted values were directionally consistent with experimental outcomes. This complementary role is important because RSM identifies significant factors and their interactions through statistical testing, whereas ANN refines predictions and minimizes residual error. Together they provide a reliable and interpretable framework for optimizing OPs extraction.

### 3.4 Chemical composition of okra polysaccharides

#### 3.4.1 Uronic acid, glucan, and total sugar contents

As shown in Table 4, the uronic acid content in polysaccharides extracted by UA-DES was significantly higher than that obtained by HWU, measuring 37.87 % and 28.80 %, respectively. These values are consistent with the ranges reported by Xiong et al. [40], who found 31.6–37.3 % uronic acid in acid-soluble pectin from okra. Since elevated uronic acid levels are frequently linked to enhanced antioxidant and immunomodulatory properties of pectic polysaccharides [41], the UA-DES extract demonstrates notable functional potential. The UA-DES method also improved glucan recovery compared with HWU, increasing α-glucan from 1.19 % to 1.51 %, β-glucan from 0.20 % to 0.50 %, and total glucan from 1.39 % to 2.01 %. This improvement indicates that UA-DES not only extracts pectin-rich fractions but also enhances the release of hemicellulosic and glucan components, which are sensitive to solvent–matrix interactions. Previous reports have shown that glucan recovery is highly dependent on both extraction method and raw material [42]. Regarding total sugar content, the UA-DES extract demonstrated markedly elevated levels across all tested concentrations compared to HWU. At a concentration of 0.50  µg/mL, the total sugar content in UA-DES reached 63.42 %, in contrast to 39.17 % in HWU. These results are consistent with the findings of Ding et al. [43], who reported 49–97 % sugar content in different OPs fractions obtained through optimized extraction methods. The substantial increase in total sugar suggests that UA-DES enhances solubilization efficiency and enriches bioactive carbohydrate fractions compared with conventional hot-water methods.

**Table 4:** Comparison of chemical composition and structural features of OPs obtained by HWU and UA-DES methods.

Contents	HWU^a^	UA-DES^b^
Total Sugar (%)	39.17 ± 0.08	63.42 ± 0.04
Uronic acid (%)	28.80 ± 30	37.87 ± 2.6
**Glucan (%)**		
α- Glucan (%)	1.19 ± 0.04	1.51 ± 0.06
β- Glucan (%)	0.20 ± 0.01	0.50 ± 0.01
Total Glucan (%)	1.39 ± 0.05	2.01 ± 0.07
**Monosaccharide composition (molar ratio)**		
Mannose	1.00	1.00
Rhamnose	30.35	15.66
Glucuronic acid	2.93	1.51
Galacturonic acid	15.93	6.47
Glucose	4.75	2.41
Galactose	40.05	29.72
Arabinose	−	1.95

a HWU: Hot water ultrasonic extraction

b UA-DES: Ultrasound-assisted DES extraction

#### 3.4.2 Monosaccharide compositions of okra polysaccharides

**Fig. 7:**
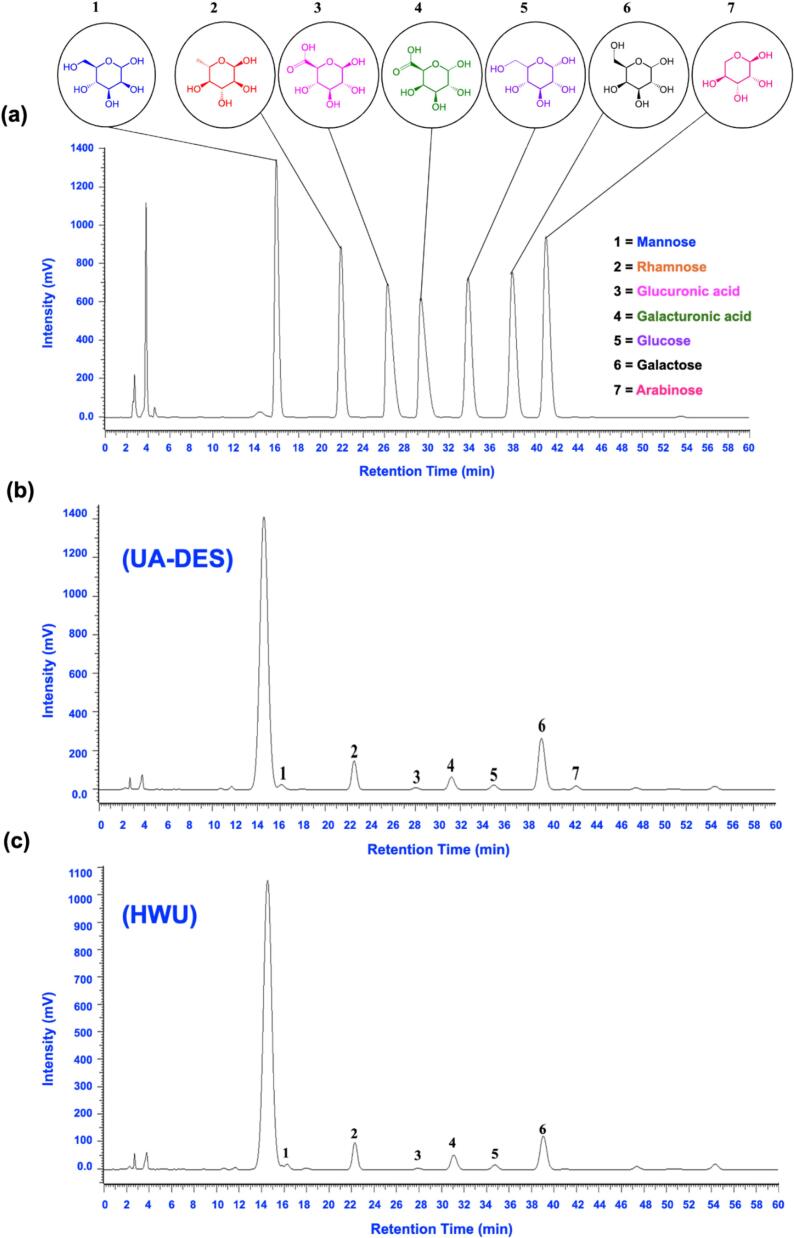
HPLC chromatograms of monosaccharide composition in OPs obtained by UA-DES and HWU. (a) Mixed monosaccharide standards (mannose, rhamnose, glucuronic acid, galacturonic acid, glucose, galactose, and arabinose). (b) OPs obtained by UA-DES. (c) OPs obtained by HWU.

Monosaccharide analysis provides essential insights into the structural characteristics of plant polysaccharides. HPLC chromatograms (Fig. 7) revealed that HWU-derived OPs consisted of mannose, rhamnose, glucuronic acid, galacturonic acid, glucose, and galactose, with molar ratios of 1.00, 30.35, 2.93, 15.93, 4.75, and 40.05, respectively (Table 4). In contrast, UA-DES-derived OPs contained mannose, rhamnose, glucuronic acid, galacturonic acid, glucose, galactose, and arabinose, with ratios of 1.00, 15.66, 1.51, 6.47, 2.41, 29.72, and 1.95. These profiles align with earlier studies on okra and other plant polysaccharides [44]. Rhamnose, galacturonic acid, and galactose were the dominant residues, consistent with polysaccharides composed primarily of rhamnogalacturonan I (RG-I) and homogalacturonan (HG) domains. The presence of galactose and arabinose further indicates RG-I side chains [45].

UA-DES extracts uniquely contained arabinose, which was absent in HWU samples, confirming that extraction methods significantly influence monosaccharide composition [46]. The detection of arabinose may be attributed to strong hydrogen bonding interactions within DESs, which facilitate cleavage of glycosidic bonds more efficiently than HWU, leading to the release of arabinose-rich side chains from pectic or hemicellulosic regions. In addition, the acidic and ionic nature of DES can interact with polysaccharide hydroxyl groups, altering monosaccharide profiles through molecular rearrangements [47]. These results suggest that UA-DES not only improves extraction efficiency but also modulates structural features, producing OPs fractions with distinct monosaccharide compositions compared with HWU.

### 3.5 Physicochemical characterization of okra polysaccharides

#### 3.5.1 FTIR and XRD analyses of okra polysaccharides

**Fig. 8:**
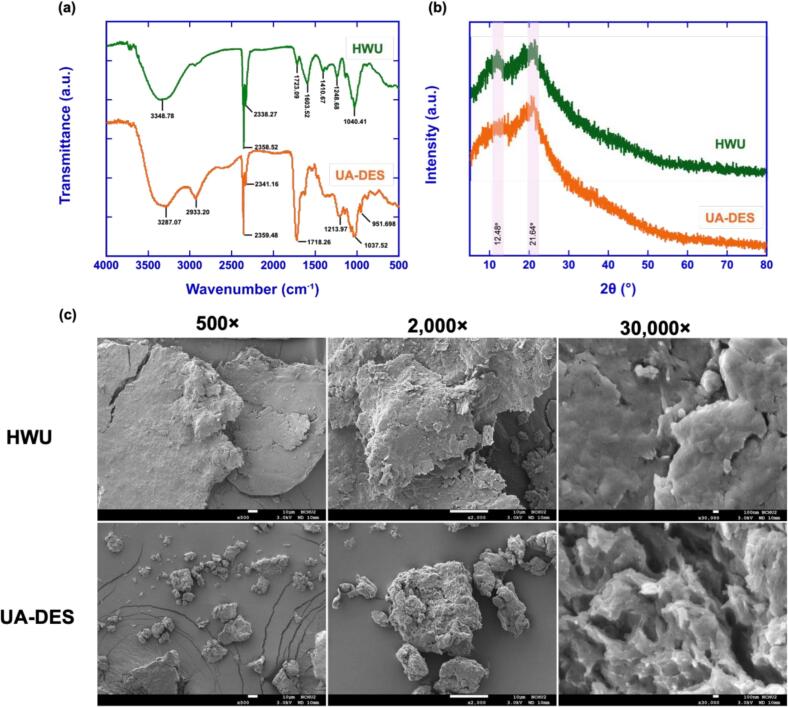
Structural characterization of OPs obtained by HWU and UA-DES. (a) FTIR spectra of OPs showing characteristic absorption bands. The broad peak around 3348 cm^−1^ corresponds to O–H stretching vibrations of hydroxyl groups, while peaks near 2933–2880 cm^−1^ are attributed to C–H stretching of aliphatic chains. The signals observed between 1700 and 1600 cm^−1^ represent C=O stretching of ester or carboxyl groups, and those in the range of 1200–1000 cm^−1^ are related to C–O–C or C–O stretching vibrations, indicating the presence of polysaccharide structures. (b) XRD patterns of OPs showing broad diffraction peaks centered at 2θ ≈ 12.5° and 21.6°, characteristic of amorphous biopolymeric materials. The disappearance of sharp crystalline peaks confirms the non-crystalline nature of OPs extracted by both HWU and UA-DES. (c) SEM micrographs showing the surface morphology of OPs obtained by HWU and UA-DES. OPs extracted by HWU (top row) and UA-DES (bottom row) were observed under different magnifications to reveal surface morphological features. Images were captured at (i) 500 × magnification, showing the overall particle structure and fracture surfaces; (ii) 2000 × magnification, highlighting the surface roughness and aggregation; and (iii) 30000 × magnification, providing detailed views of the microstructural porosity and fine surface texture.

The FTIR spectra of OPs extracted by HWU and UA-DES (Fig. 8a) showed generally similar profiles, yet UA-DES samples displayed more distinctive absorption features. A strong and broad band at 3287.07 cm^−1^ indicated O–H stretching vibration, while the peak at 2933.20 cm^−1^ corresponded to C–H stretching of CH_3_ and CH_2_ groups. The absorption at 1718.26 cm^−1^ represented C=O stretching [48]. The band at 1213.97 cm^−1^, assigned to asymmetric and symmetric stretching of carboxylic acid groups, confirmed the presence of uronic acids, which is consistent with the monosaccharide analysis [49]. A strong band at 1037.52 cm^−1^, caused by overlapping C–O–C and C–O–H stretching, implied the presence of pyranose rings [50]. Notably, this feature was absent in HWU samples, suggesting that UA-DES better preserved or enhanced structural motifs relevant to bioactivity.

XRD analysis further confirmed the amorphous nature of OPs (Fig. 8b). Both HWU and UA-DES-derived polysaccharides showed broad diffraction peaks near 12° and 22° with low intensity, characteristic of amorphous structures. Amorphous configurations are known to increase solubility and may enhance bioactivity in functional applications [51]. Our observations are consistent with Wang et al*.* [48] and Ma et al*.* [52], who also identified the amorphous characteristics of okra pericarp polysaccharides. The similarity of the diffraction profiles suggests that UA-DES maintained the amorphous state typical of OPs, while additional functional group preservation detected by FTIR supports UA-DES as a more structurally favorable method than HWU.

#### 3.5.2 Morphology of okra polysaccharides

SEM images (Fig. 8c) revealed distinct morphological differences between HWU and UA-DES-derived OPs. HWU extraction yielded aggregated, compact particles with smooth, non-porous surfaces (Fig. 8c. top row), indicating limited cell wall disruption. By contrast, UA-DES extraction produced fragmented and dispersed particles with rough, porous surfaces (Fig. 8c, bottom row). The porous structure, characterized by visible uniform pores, corresponds to greater surface area and is likely to facilitate solubilization and enhance functional properties. These findings agree with Zhang et al. [53], who reported that DES-extracted polysaccharides from *Lentinus edodes* displayed filamentation, high porosity, and rough surfaces, associated with improved antioxidant activity, whereas HWU products remained compact and smooth. The presence of porous, roughened morphology in UA-DES-derived OPs implies that cavitation and solvent interactions promote microstructural loosening, which enhances accessibility of bioactive moieties and supports superior functional performance. Overall, the combined FTIR, XRD, and SEM results indicate that UA-DES not only maintains the amorphous nature of OPs but also enhances preservation of functional groups and produces a porous morphology, all of which contribute to improved solubility and bioactivity compared with HWU.

### 3.6 Antioxidant activity of okra polysaccharides

**Fig. 9:**
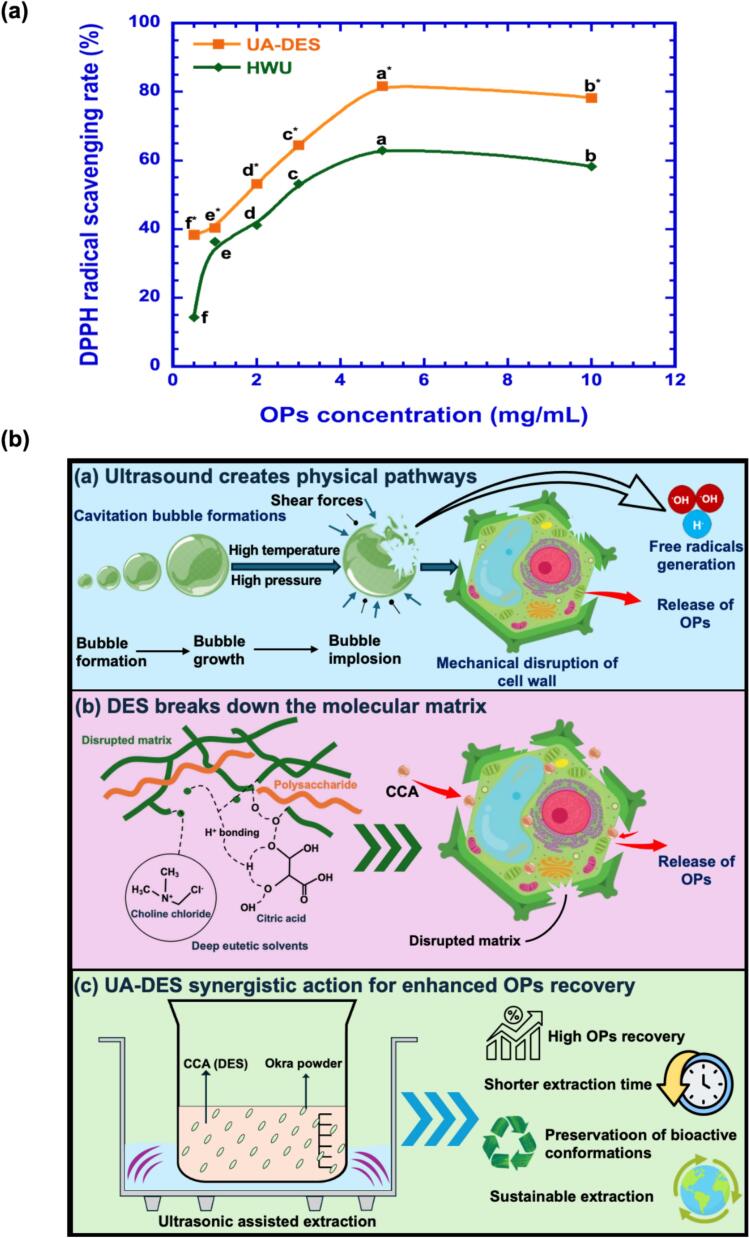
(a) DPPH radical scavenging activity of OPs obtained by UA-DES and HWU. OPs samples extracted by UA-DES and HWU were tested at concentrations ranging from 1 to 10 mg/mL. DPPH concertation was 0.1 mM. Data are expressed as mean ± SD (n = 3). Different letters above the data points indicate significant differences among concentrations within the same extraction method, as determined by one-way ANOVA followed by Tukey’s post hoc test. Statistical significance was considered at *P* < 0.05. (b) Proposed mechanisms of deep eutectic solvent coupled with ultrasound-assisted (UA-DES) extraction of OPs. In this system, choline chloride/citric acid-based DES penetrates the plant matrix and disrupts hydrogen-bonding networks, while ultrasound cavitation generates bubbles and shear forces that mechanically break cell walls. The combined effects facilitate solvent infiltration and promote the release of intracellular polysaccharides and phytochemicals. The citric acid component contributes to pH modulation and chelation of metal ions, thereby enhancing polysaccharide solubilization and extraction efficiency. Additionally, free radical generation during cavitation may further increase solvent penetration and extraction performance.

The antioxidant activity of OPs extracted by HWU and UA-DES was assessed using the DPPH^•^ radical scavenging assay at different concentrations (0.5, 1.0, 2.0, 3.0, 5.0, and 10.0 mg/mL) (Fig. 9a). At 5 mg/mL, UA-DES-derived OPs exhibited markedly higher scavenging activity (81.69 %) compared with HWU-derived OPs (62.94 %). This difference was statistically significant (p < 0.05). This indicates that the UA-DES system not only improves extraction efficiency but also yields products with stronger antioxidant potential. Compared to the conventional HWU process, UA-DES produced OPs with nearly double the yield (23.56 % vs. 12.11 %) while reducing extraction time from 240 to 120 min and energy consumption from 1.52 to 0.38 kWh. This improved energy economy, combined with the enhanced bioactivity, demonstrates that UA-DES extraction provides both functional and economic advantages. The results summarized in Tables S3 and S5 further support this trend, showing that UA-DES achieves higher yields and bioactivities than other plant polysaccharide extractions.

To strengthen the antioxidant evaluation, ABTS (2,2′-azino-bis (3-ethylbenzothiazoline-6-sulfonic acid radical ion)), FRAP (ferric reducing antioxidant power), TFC (total flavonoid contents), and TPC (total phenolic contents) analyses were further conducted, and the results were summarized in Table S5. These assays provided a broader view of the radical-scavenging and electron-donating capacities of OPs. Across all indicators, UA-DES-extracted OPs consistently demonstrated higher antioxidant performance than HWU-derived OPs. The values obtained from UA-DES also aligned well with or surpassed those reported in literature (Table S5) for okra polysaccharides extracted by acidic media, microwave-assisted extraction, and ultrasound-assisted extraction. This comparison confirms that the improvement observed in the present study is not limited to DPPH^•^ activity but is supported by multiple assessment methods that reflect both phenolic-associated and polysaccharide-associated antioxidant mechanisms. The enhanced performance of UA-DES OPs therefore reflects a genuine strengthening of antioxidant potential rather than a method-specific artifact.

The improved DPPH^•^ scavenging ability of UA-DES OPs can be attributed to alterations in monosaccharide composition and structural modifications induced by DES. Robust hydrogen-bonding interactions within DES promote cleavage of glycosidic bonds and can generate new sugar residues such as arabinose, in addition to inducing epimerization of glucose into mannose and galactose [54]. These structural changes introduce diverse hydroxyl groups and modify molecular configurations, thereby enhancing the ability of OPs to donate hydrogen atoms or electrons for free radical scavenging [55]. The unique presence of arabinose in UA-DES-derived OPs, absent in HWU fractions, provides further mechanistic evidence of enhanced reactivity due to DES-mediated structural modulation. The dose-dependent increase in DPPH^•^ activity observed in this study is consistent with earlier reports on OPs [52]. At higher concentrations, polysaccharides exhibit greater radical scavenging capacity by providing more active hydroxyl donors [56]. Together, these findings confirm that UA-DES not only improves extraction yield but also enriches polysaccharide fractions with enhanced antioxidant properties, thereby establishing UA-DES as an efficient and functional extraction approach.

### 3.7 UA-DES-based okra polysaccharides (OPs) extraction mechanism

As shown in Fig. 9b, UA-DES extraction of OPs relies on the synergistic effects of acoustic cavitation and the unique physicochemical properties of DESs to enhance extraction efficiency. Ultrasonic waves passing through the DES medium create alternating high- and low-pressure cycles, which induce the formation, growth, and collapse of cavitation bubbles. These implosions produce extreme localized conditions with transient temperatures up to 5,000 K and pressures around 1,000 atm [57]. The collapse of cavitation bubbles generates strong shear forces, shock waves, and high-speed microjets (200–700 m/s), mechanically disrupting plant cell walls and releasing polysaccharides trapped in the intracellular matrix [58]. Following cell wall disruption, DESs play a critical role in polysaccharide solubilization. Hydrogen bond donors (HBDs) and acceptors (HBAs) in DESs enhance solvent penetration by breaking intermolecular hydrogen bonds within the plant matrix and adjusting polarity to facilitate polysaccharide release [18], [59]. This dual effect of physical cell wall rupture and chemical solubilization differentiates UA-DES from conventional hot-water extraction [57], [60].

The acidic pH of citric acid–based DESs further promotes extraction efficiency. Acidic conditions neutralize pectic elements, disrupt calcium-mediated crosslinking, weaken cellulose–pectin interactions, and enhance hydration, thereby loosening the plant cell wall [61]. These changes improve polysaccharide mobility and significantly increase extraction yield. Previous reports confirm that acidic DESs achieve maximum dissolution of polysaccharides [34], [55]. Our results also demonstrate that citric acid–based DESs provide superior efficiency compared with sugar- or alcohol-based DESs, highlighting solvent composition as a decisive factor. In addition to physicochemical modifications, cavitation in aqueous or hydroxyl-rich DES systems can generate hydroxyl (^•^OH) and hydrogen (H^•^) radicals, which facilitate loosening of polysaccharide complexes without excessive degradation under optimized conditions [18]. Non-cavitation effects such as acoustic streaming, microstreaming, and localized pressure gradients also enhance solvent diffusion and mixing, which is particularly beneficial in viscous DES systems. By optimizing ultrasound parameters (power, temperature, duration), solvent composition, pH, and liquid–solid ratio, UA-DES achieves efficient cell wall disruption, accelerated mass transfer, and preservation of bioactive compounds. This holistic integration of mechanical and chemical mechanisms makes UA-DES an efficient, eco-friendly, and sustainable strategy for producing high-quality polysaccharides.

## 4 Conclusions

Conventional extraction approaches for okra polysaccharides (OPs) are often limited by low extraction efficiency, long processing times, and insufficient preservation of structural and functional integrity. These limitations underscore the need for a more sustainable and effective extraction strategy that can enhance yield and preserve bioactivity. This study demonstrates that the ultrasound-assisted deep eutectic solvent (UA-DES) system, utilizing choline chloride–citric acid, significantly enhances the extraction performance of OPs. Optimization identified sonication time and liquid–solid ratio as key parameters, while artificial neural network (ANN) modeling outperformed response surface methodology in predictive accuracy. Under optimized conditions, the UA-DES method produced higher OPs yields and more potent antioxidant activity compared with hot-water extraction. Structural characterization further confirmed the preservation of pyranose configurations, amorphous crystallinity, and a porous microstructure, which are collectively associated with improved solubility and functional potential. These results validate the synergistic effect of combining ultrasound with DESs, offering enhanced mass transfer and solubilization, and demonstrating a promising platform for producing structurally robust and bioactive polysaccharides. The optimized UA-DES approach provides an efficient, reproducible, and environmentally friendly method suitable for applications in food, nutraceutical, and functional ingredient development. This study was conducted at the laboratory scale, focusing primarily on the antioxidant properties. Additionally, while the UA-DES system shows strong extraction performance, solvent recyclability and long-term operational feasibility were not evaluated in this work. Future studies should investigate pilot-scale or industrial-scale processing, evaluate the recyclability and environmental impacts of DES, explore the additional biological activities of extracted OPs, and assess the versatility of UA-DES extraction for other plant polysaccharides. Overall, the optimized UA-DES method provides a sustainable and high-performing extraction strategy for okra polysaccharides, supporting the broader development of green technologies for bioactive compound recovery and laying the foundation for future industrial applications.

## CRediT authorship contribution statement

**Muhammad Imran:** Writing – original draft, Investigation, Formal analysis, Data curation. **Chih-Huang Weng:** Writing – review & editing, Validation. **Girma Sisay Wolde:** Writing – review & editing, Validation. **Ying-Chen Chen:** Writing – review & editing. **Yi-Jin Wu:** Writing – review & editing. **Shang-Ming Huang:** Writing – review & editing, Revision, Review & editing, Visualization, Validation, Supervision, Resources, Conceptualization. **Yao-Tung Lin:** Writing – review & editing, Visualization, Validation, Supervision, Resources, Project administration, Funding acquisition, Conceptualization.

## Credit authorship contribution statement

Muhammad Imran contributed to formal analysis, data curation, investigation, and writing of the original draft. Chih-Huang Weng was involved in data curation, writing of the original draft, and writing, review, and editing. Girma Sisay Wolde contributed to validation as well as writing, review, and editing. Ying-Chen Chen was responsible for methodology and participated in writing, review, and editing. Yi-Jin Wu contributed to writing, review, and editing. Shang-Ming Huang contributed to conceptualization, resources, visualization, project administration, and investigation, as well as writing, reviewing and editing of the original draft, and responsible for the revision. Yao-Tung Lin contributed to conceptualization, visualization, supervision, and funding acquisition, and was also responsible for writing, review, and editing.

## Declaration of competing interest

The authors declare that they have no known competing financial interests or personal relationships that could have appeared to influence the work reported in this paper.
