# Supplementary material for: Ultrasound-Assisted Deep Eutectic Solvent-Based Extraction of Polysaccharides from Okra: Optimization by Response Surface Methodology and Artificial Neural Network Modeling
Source: Ultrason Sonochem. 2025 Dec 9;124:107715. doi: 10.1016/j.ultsonch.2025.107715 (PMC12769841; doi:10.1016/j.ultsonch.2025.107715)
Supplement: Supplementary Data 1 [file mmc1.docx]

**Supplementary Data**

## **2. Materials and methods: 2.1 Determination of ABTS (2,2’-azino-bis (3-ethylbenzo- thiazoline-6-sulfonic acid radical ion)) of okra polysaccharide**

ABTS⁺ radical scavenging activity was determined according to the ABTS⁺ decolorization assay with some modifications by Li et al. (2012). The ABTS⁺ working solution was prepared by mixing 7 mM ABTS with 140 mM potassium persulfate at a 5 mL:88 µL ratio and allowing the mixture to react in the dark at room temperature for 20 h to form the ABTS⁺ radical cation. The ABTS⁺ solution was further diluted with 95% ethanol to a value of 0.70 ± 0.02 at 734 nm before use. Trolox was used as the standard. A 1 mg/mL Trolox stock solution was prepared in 95% ethanol and further diluted into a series of concentrations from 0 to 0.04 mg/mL. To run the assay, 50 µL of each standard or sample was added into a 96-well microplate, followed by the addition of 200 µL of diluted ABTS⁺ solution. The plate was subjected to shaking at 250 rpm for 10 s with a temperature at 30°C and incubation for 6 min in the dark. The results were read at 734 nm. The comparable, the reaction corresponded to the ratio of 1 mL sample to 4 mL ABTS⁺ solution in the conventional tube-based method.

$ABTS radical savenging rate \left( \% \right)=(1-\frac{A_{s}-A_{b}}{A_{0}})\times10$0 1

Where A_s_, A_b_, A_0_ represented the absorbance of the sample solution (sample and ABTS), background solution (sample and deionized water), and blank solution (ABTS and deionized water), respectively.

## **2.2 Ferric reducing antioxidant power (FRAP)**

The FRAP value of okra polysaccharides (OPs) was assessed using the method described by Benzie & Strain, (1996). The ferrous ammonium sulfate standard (1 mg/mL) solution was prepared using double-distilled water (DDW) and serially diluted (0-500 μg/mL). The FRAP reagent was freshly mixed using 4 mM TPTZ, 80 mM ferric chloride dissolved in DDW, and 1 mM sodium acetate buffer (pH 3.6) at a 1:1:1 ratio. The OPs samples were dissolved at 10 mg/mL concentration and serially diluted (0.156-10 mg/mL). The mixture of 50 μL standard or sample and 150 μL FRAP reagents was pipetted into each well of a 96-well plate and assessed at 593 nm after 5 min at room temperature. The FRAP values were obtained using a standard curve and quantitatively expressed as mg FeSO_4_/g sample.

## **2.3 Total flavonoid content (TFC) of okra polysaccharide**

The TFC of OPs was assessed employing the aluminum chloride colorimetric assay by Chang et al. (2002). Quercetin (1 mg/mL dissolved in 95% ethanol) was employed as standard and progressively diluted (0-500 μg/mL). The analytical reagent solution consisted of equal parts 10% aluminum chloride 6H_2_O and 1M potassium acetate. A 96-well plate assay consisted of 25-μL OPs sample and standard solution, 75-μL ethanol, 10-μL analytical reagent solution, and 140-μL DDW. The mixture was agitated (250 rpm) for 10 min and allowed to stand in the dark at room temperature for 30 min. The resulting absorption was read at 420 nm, and results were quantified as mg/mg Quercetin Equivalent per gram OPs sample.

## **2.4 Total phenolic content (TPC) of okra polysaccharide**

The TPC assay of OPs was estimated using Folin-Ciocalteu method Singleton et al. (1999). The standard gallic acid (1 mg/mL DDW) concentration range was 0 to 500 μg/mL. The mixture consisted of 20 μL OPs sample/standard and 75 μL DDW, 15 μL Folin-Ciocalteu reagent, and 175 μL sodium carbonate (7.5%) on a 96-well plate. The mixture was shaken at 250 rpm for 3 min. This was immediately followed by 30 min of darkness. Absorbance at 750 nm was noted, and results were calculated as mg gallic acid equivalent/g sample.

Table S1. The viscosity, pH, and conductivity of each DES formulation

| **DES** | **Viscosity (mPa.s^-1^)** | **pH** | **EC (mS/cm)** |
| --- | --- | --- | --- |
| **ChCl : Citric acid** | **22.40 ± 0.36** | 0.435 | 10.41 |
| **ChCl : Urea** | **24.33 ± 0.80** | 5.699 | 38.50 |
| **ChCl : Glycerol** | **29.87 ± 0.95** | 5.450 | 16.22 |
| **ChCl : Malic acid** | **45.63 ± 1.29** | 0.607 | 22.68 |
| **ChCl : Ethylene glycol** | **32.93 ± 1.94** | 1.464 | 14.36 |
| **ChCl : 1,4-butanediol** | **30.33 ± 1.08** | 3.693 | 11.31 |

Table S2. Experimental independent variables and coded levels for ultrasound-assisted DES extraction of OPs optimization

| **Independent variables** | **Coded levels** | | |
| --- | --- | --- | --- |
|  | **-1** | **0** | **+1** |
| **Sonication time (h)** | 1.0 | 2.0 | 3.0 |
| **Temperature (^°^C)** | 60 | 70 | 80 |
| **Sonication power (W)** | 114 | 190 | 266 |
| **L-S (mL/g)** | 40 | 50 | 60 |

Table S3. Comparison of literature studies for the plant polysaccharide extraction from different plant sources

| **Polysaccharide source** | **Extraction method** | **Sonication time (min)** | **Temperature (°C)** | **Sonication Power (W)** | **L-S (mL/g)** | **Polysaccharide yield (%)** | **Energy consumption (kWh)** | **Reference** |
| --- | --- | --- | --- | --- | --- | --- | --- | --- |
| *Okra* | UA-DES | 120 | 80 | 190 | 60 | 23.56 | 0.38 | This Study |
| *Okra* | HWU | 240 | 80 | 380 | 60 | 12.11 | 1.52 | This Study |
| *Radix Bupleuri* | UA-DES | 60 | 57 | 400 | 30 | 10.10 | 0.40 | (Wang & Li, 2022) |
| *Dandelion* | UA-DES | 60 | 58 | 410 | 25 | 6.85 | 0.41 | (Zhang et al., 2024) |
| Indocalamus tessellatus | UA-DES | 60 | 64 | 500 | 17 | 0.68 | 1.00 | (Zhang et al., 2022) |
| *Acanthopanax senticosus* | UA-DES | 129 | 60 | 240 | 31 | 3.55 | 0.52 | (Xue et al., 2024) |
| *Rosa roxburghii Tratt.* | UA-DES | 99 | - | 240 | 52 | 3.40 | 0.40 | (Wen & Chen, 2025) |
| *Porphyra haitanensis* | UA-DES | 120 | 80 | 200 | 60 | 4.42 | 0.40 | (Hu et al., 2025) |
| *Quinoa* | UAE-Alkali | 70 | 62 | 386 | 28 | 7.13 | 0.45 | (Li et al., 2025) |
| Ulva rigida | UAE- Water bath | 126 | 68 | - | 68 | 13.22 | - | (Akbal et al., 2024) |
| Vigna radiata | Alkaline-NaOH | 180 | 90 | - | 20 | 4.08 | - | (Ding et al., 2025) |
| Chlorella | Freeze thaw-NaCl | 120 | -20 | - | 20 | 2.30 | - | (Zhou et al., 2025) |
| *Perilla frutescens* | MAE-Water | 210 | 80 | 700 | 20 | 3.96 | 2.45 | (Liu et al., 2025) |

Table S4. Validation of optimal conditions for ultrasound-assisted DES extraction of OPs by comparing experimental values with predicted results from RSM and ANN models

|  | **Independent variable** | | | | | |  | **RSM** | | **ANN** | |
| --- | --- | --- | --- | --- | --- | --- | --- | --- | --- | --- | --- |
| **Parameters** | | | **Time (h)** | **Temperature (^°^C)** | **Power (W)** | **L-S (mL/g)** | **Actual** | **Pred.** | **Res.** | **Pred.** | **Res.** |
| **OPs yield (%)** | | **Predicted** | 2.39 | 80.7 | 193 | 60.5 | 23.56 | 23.47 | 0.09 | 23.58 | 0.02 |
|  |  | **Actual** | 2 | 80 | 190 | 60 |  |  |  |  |  |
| **DPPH**^•^ **scavenging activity (%)** | | **Predicted** | 2.39 | 80.7 | 193 | 60.5 | 80.75 | 79.76 | 0.99 | 80.90 | 0.15 |
|  |  | **Actual** | 2 | 80 | 190 | 60 |  |  |  |  |  |

Pred: Predicted, Res: Residual

Table S5. Comparison of antioxidant activities (DPPH, ABTS, and FRAP assays), total flavonoid content (TFC), and total phenolic content (TPC) of okra polysaccharides obtained using different extraction methods

| **Extraction method** | **DPPH**  **(%)** | **ABTS**  **(%)** | **FRAP**  **(μmol Fe²⁺/g)** | **TFC**  **(mg antioxidant/g)** | **TPC**  **(mg GAE/g)** | | **References** |
| --- | --- | --- | --- | --- | --- | --- | --- |
| UA-DES | 82 | 78 | 172 | 10 | 1.85 | This study | |
| HWU | 69 | 57 | 101 | 4.0 | 0.63 | This study | |
| Acidic | 62 | 76 | - | - | - | (Xiong et al., 2021) | |
| HWU | 79 | - | 169 | - | - | (Wang et al., 2020) | |
| HWU | 85 | - | - | 18.72 | 49.93 | (Gemede et al., 2018) | |
| HWU | - | - | - | 20.43 | - | (Nie et al., 2019) | |
| HWU | 69 | - | - | - | - | (Ma et al., 2021) | |
| UAE | 79 | - | - | - | - | (Wang et al., 2018) | |
| MAE | - | 69 | 30 | - | - | (Yuan et al., 2019) | |
| UAE | - | 75 | - | - | - | (Nie et al., 2020) | |
| UAE | 58 | 99 | - | 4.10 | 1.02 | (Olawuyi & Lee, 2021) | |
| UAE |  | 61 | 65 | - | - | (Li et al., 2022) | |

**
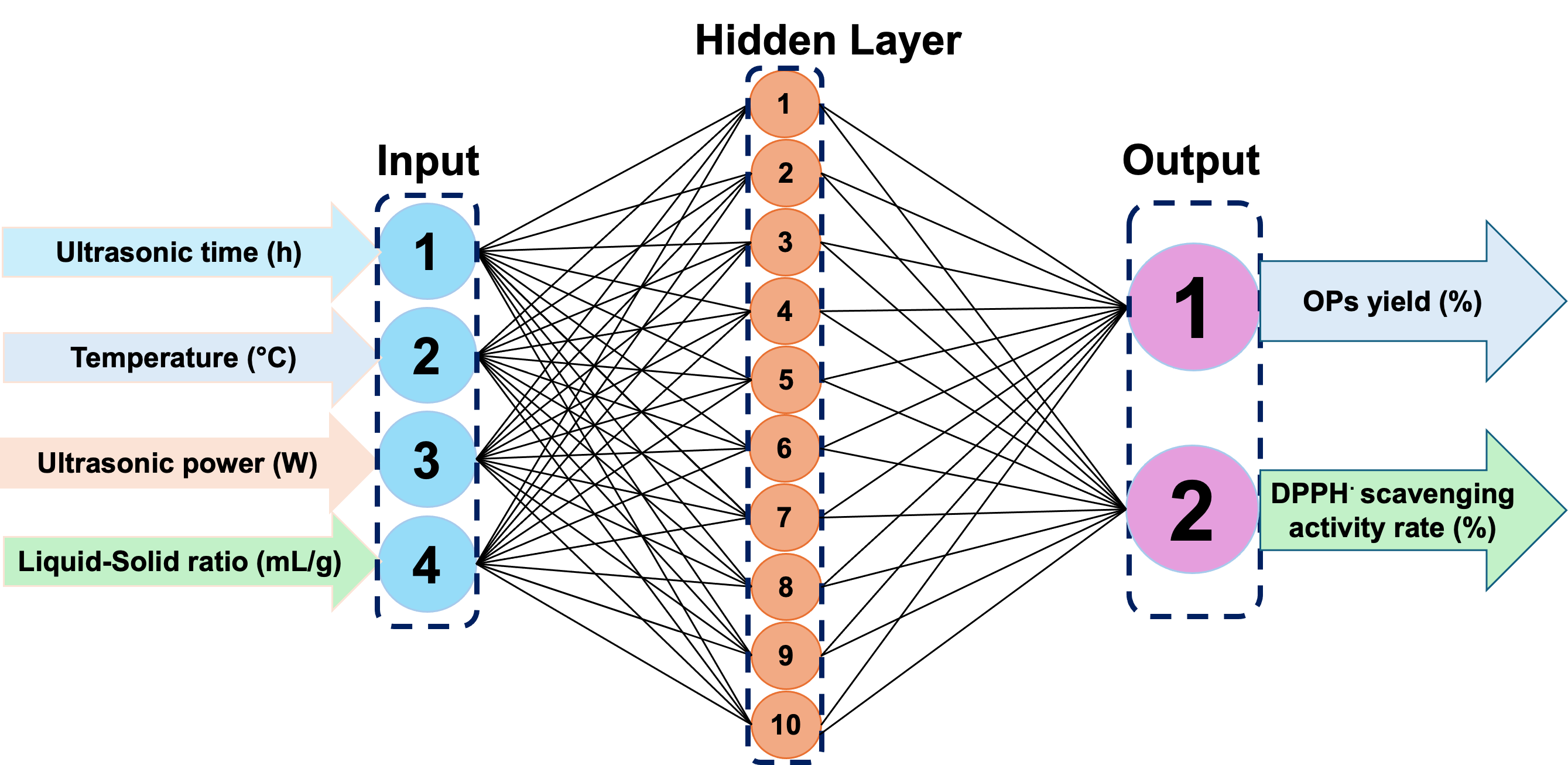
**

**Fig. S1.** Schematic structure illustrating the application of a neural network model for predicting the shielding performance of OPs yield and its DPPH^•^ radical scavenging. The model architecture includes input parameters, hidden layers, and output responses to demonstrate the predictive framework.


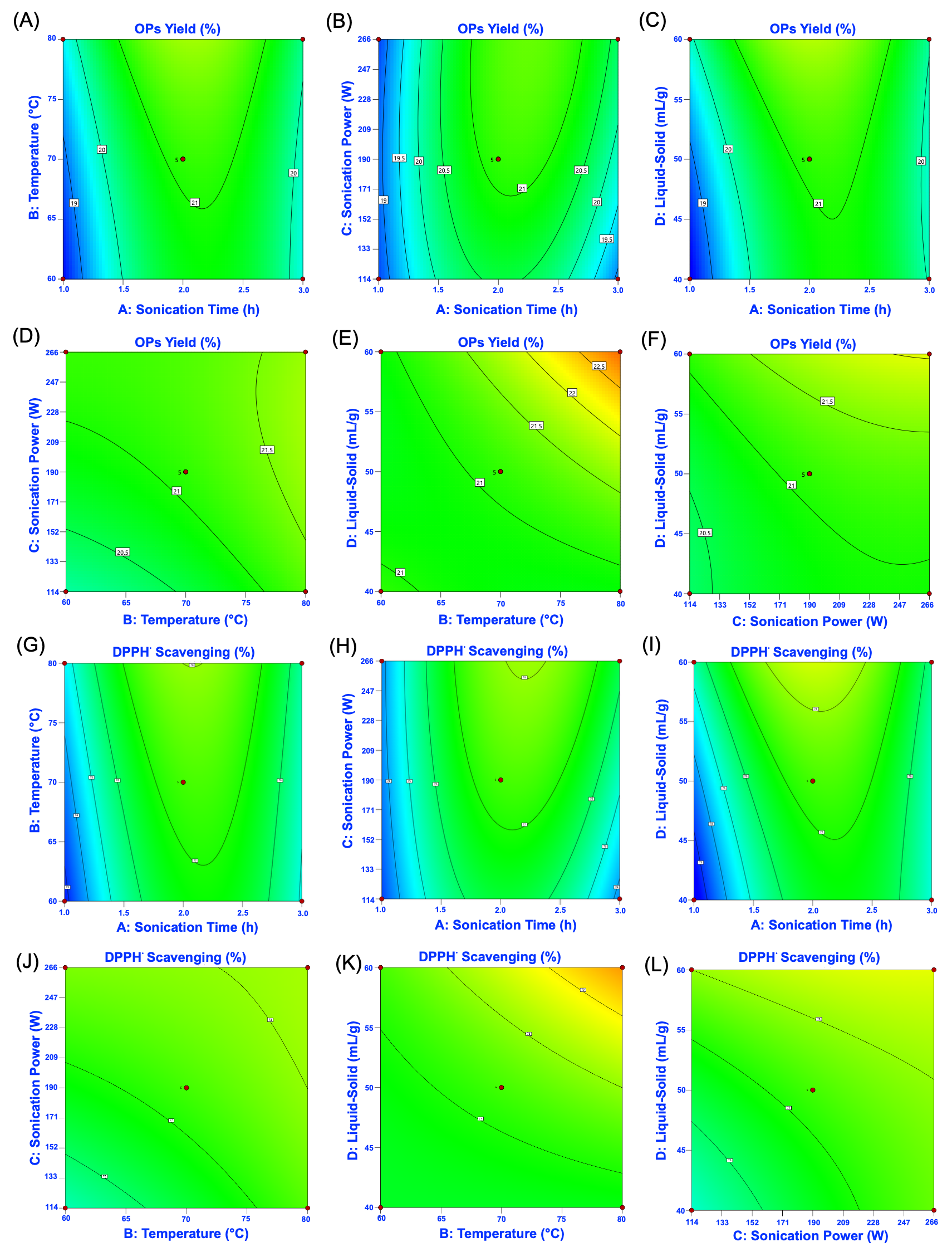


**Fig. S2.** Response contour plots for the OPs yield (a-f) and DPPH^•^ scavenging activity (g-l).


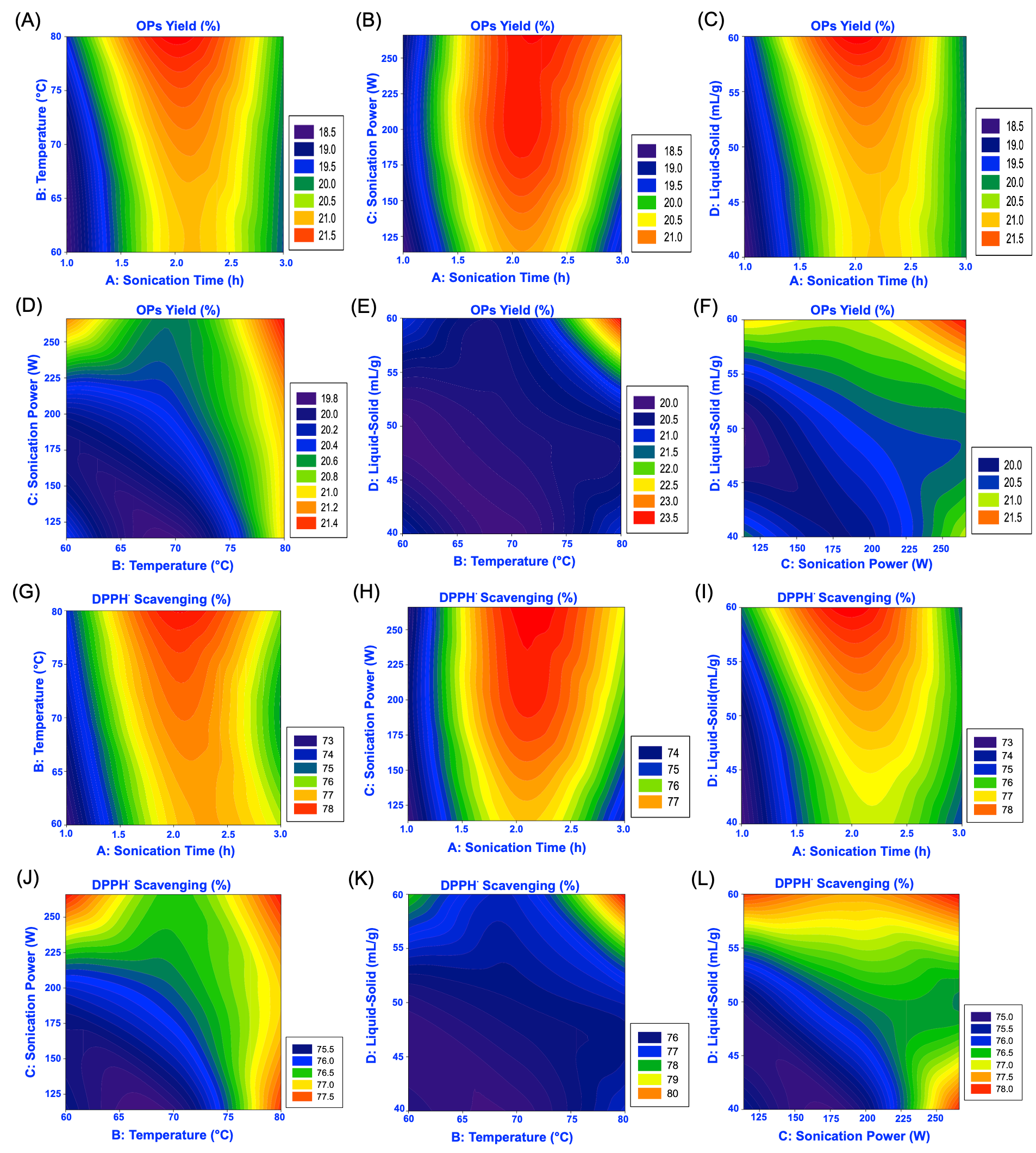


**Fig. S3.** ANN contour plots for the OPs yield (a-f) and DPPH^•^ scavenging activity (g-l).


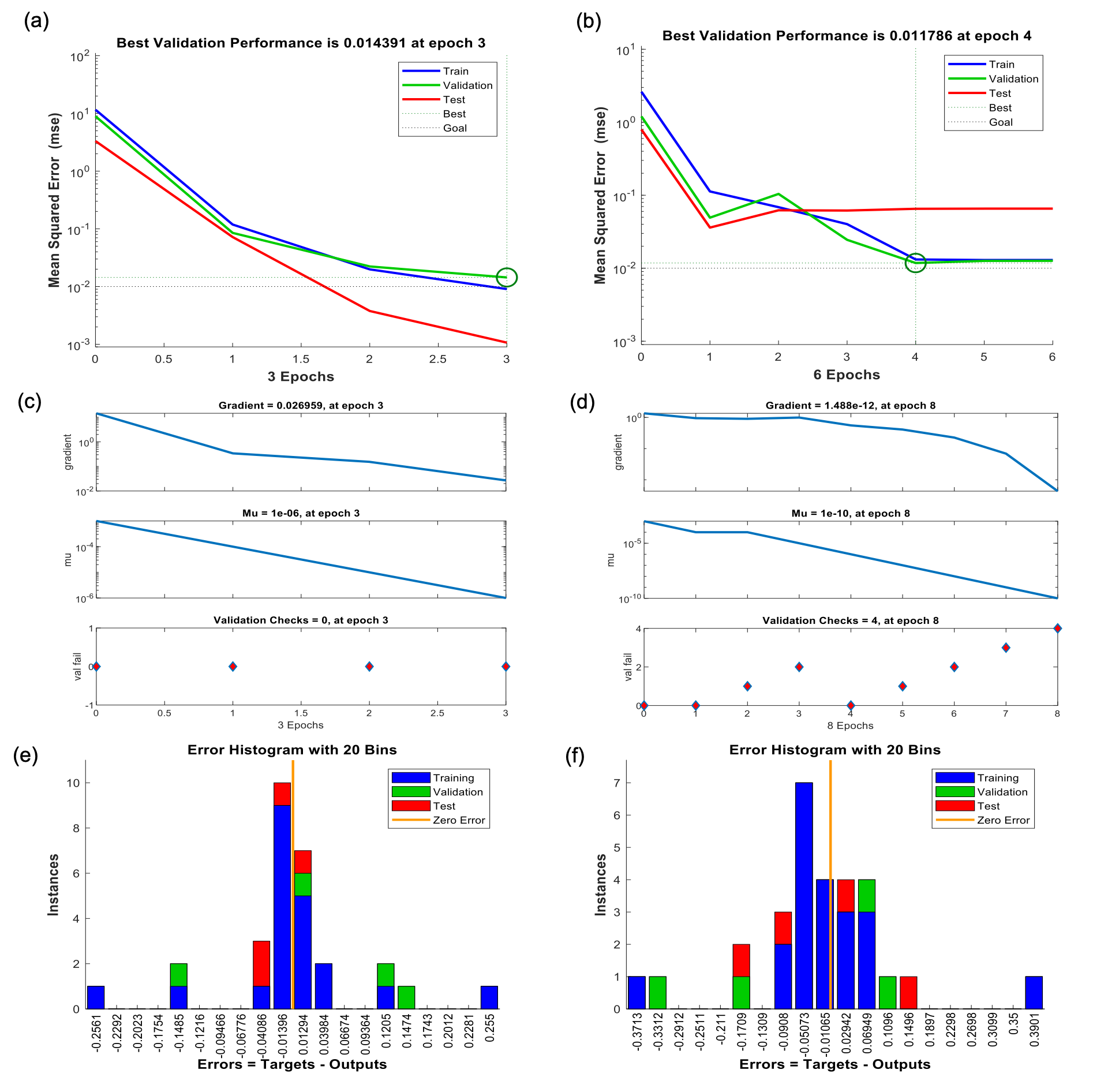
 **Fig. S4.** ANN model for the MSE for different data sets for the best validation performance (a), training state (b), and error histogram (c) for the OPs yield and for the DPPH^•^ scavening activity (d-f), respectively.


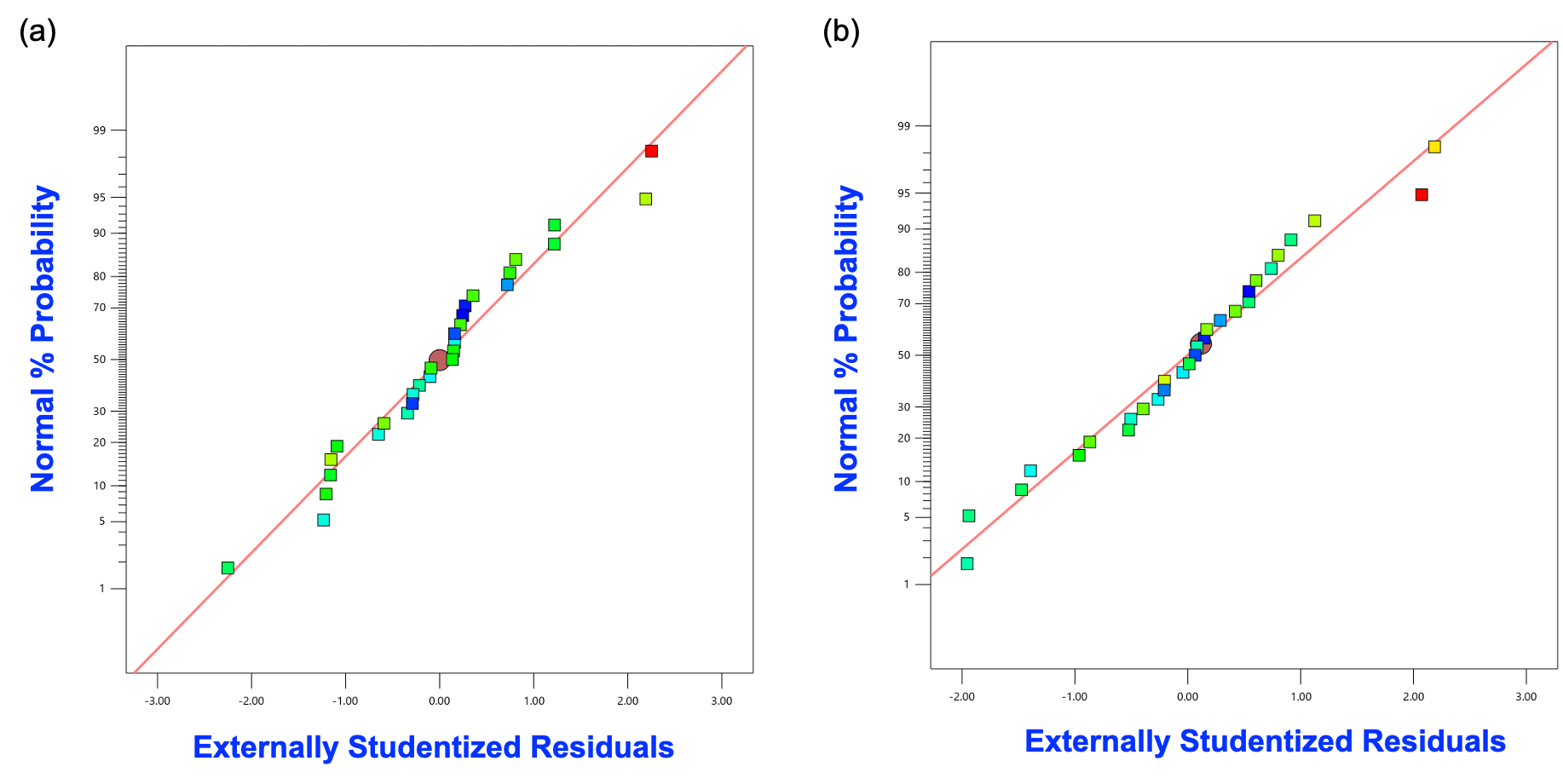


**Fig. S5.** RSM based normal % probablity of externally studentized residuals of OPs yield (a), and DPPH^•^ scavenging activity (b).

# References

Akbal, A., Şahin, S., & Güroy, B. (2024). Optimization of ultrasonic-assisted extraction of polysaccharides from Ulva rigida and evaluation of their antioxidant activity. *Algal Research*, *77*, 103356. <https://doi.org/https://doi.org/10.1016/j.algal.2023.103356>

Benzie, I. F., & Strain, J. J. (1996). The ferric reducing ability of plasma (FRAP) as a measure of “antioxidant power”: the FRAP assay. *Analytical biochemistry*, *239*(1), 70–76.

Chang, C.-C., Yang, M.-H., Wen, H.-M., & Chern, J.-C. (2002). Estimation of total flavonoid content in propolis by two complementary colorimetric methods. *Journal of food and drug analysis*, *10*(3).

Ding, X., Li, T., Zhao, J., Khalid, W., Fan, M., Qian, H., Li, Y., & Wang, L. (2025). Effect of various extraction methods on the physicochemical properties, antioxidant, and anti-inflammatory activities of mung bean (Vigna radiata L.) skin polysaccharides. *International Journal of Biological Macromolecules*, *311*, 143969. <https://doi.org/https://doi.org/10.1016/j.ijbiomac.2025.143969>

Gemede, H. F., Haki, G. D., Beyene, F., Rakshit, S. K., & Woldegiorgis, A. Z. (2018). Indigenous Ethiopian okra (Abelmoschus esculentus) mucilage: A novel ingredient with functional and antioxidant properties. *Food Science & Nutrition*, *6*(3), 563–571.

Hu, W., Liu, B., & Wen, Y. (2025). Optimization of Ultrasound‐Assisted Deep Eutectic Solvent Extraction and Bioactivities of Polysaccharide from Porphyra haitanensis. *Starch‐Stärke*, *77*(1), 2300272.

Li, H., Li, C., Xu, Y., Cao, H., Wang, X., & He, J. (2025). Ultrasonic-assisted alkali extraction of quinoa polysaccharides: Yield and structural characterization. *Journal of Cereal Science*, *122*, 104108. <https://doi.org/https://doi.org/10.1016/j.jcs.2025.104108>

Li, W., Li, J., Wang, J., He, Y., Hu, Y.-C., Wu, D.-T., & Zou, L. (2022). Effects of various degrees of esterification on antioxidant and immunostimulatory activities of okra pectic-polysaccharides. *Frontiers in Nutrition*, *9*, 1025897.

Li, X., Lin, J., Gao, Y., Han, W., & Chen, D. (2012). Antioxidant activity and mechanism of Rhizoma Cimicifugae. *Chemistry Central Journal*, *6*(1), 140.

Liu, X., Li, Z., Xu, L., Pan, Y., Li, W., Ran, X., Meng, Z., Zhang, J., & Xing, Y. (2025). Optimization of Polysaccharide Extraction Technology From Perilla frutescens Leaves and Its α‐Glucosidase Inhibition Activity. *Journal of Food Biochemistry*, *2025*(1), 8749940.

Ma, L.-Y., Xu, R., Lin, H.-F., Xie, M.-Y., Nie, S.-P., & Yin, J.-Y. (2021). Structural characterization and antioxidant activities of polysaccharides from okra (Abelmoschus esculentus (L.) Moench) pericarp. *Bioactive Carbohydrates and Dietary Fibre*, *26*, 100277.

Nie, X.-R., Fu, Y., Wu, D.-T., Huang, T.-T., Jiang, Q., Zhao, L., Zhang, Q., Lin, D.-R., Chen, H., & Qin, W. (2020). Ultrasonic-assisted extraction, structural characterization, chain conformation, and biological activities of a pectic-polysaccharide from okra (Abelmoschus esculentus). *Molecules*, *25*(5), 1155.

Nie, X.-R., Li, H.-Y., Du, G., Lin, S., Hu, R., Li, H.-Y., Zhao, L., Zhang, Q., Chen, H., & Wu, D.-T. (2019). Structural characteristics, rheological properties, and biological activities of polysaccharides from different cultivars of okra (Abelmoschus esculentus) collected in China. *International Journal of Biological Macromolecules*, *139*, 459–467.

Olawuyi, I. F., & Lee, W. Y. (2021). Structural characterization, functional properties and antioxidant activities of polysaccharide extract obtained from okra leaves (Abelmoschus esculentus). *Food chemistry*, *354*, 129437.

Singleton, V. L., Orthofer, R., & Lamuela-Raventós, R. M. (1999). [14] Analysis of total phenols and other oxidation substrates and antioxidants by means of folin-ciocalteu reagent. In *Methods in enzymology* (Vol. 299, pp. 152–178). Elsevier.

Wang, C., Yu, Y.-B., Chen, T.-T., Wang, Z.-W., & Yan, J.-K. (2020). Innovative preparation, physicochemical characteristics and functional properties of bioactive polysaccharides from fresh okra (Abelmoschus esculentus (L.) Moench). *Food chemistry*, *320*, 126647.

Wang, K., Li, M., Wen, X., Chen, X., He, Z., & Ni, Y. (2018). Optimization of ultrasound-assisted extraction of okra (Abelmoschus esculentus (L.) Moench) polysaccharides based on response surface methodology and antioxidant activity. *International Journal of Biological Macromolecules*, *114*, 1056–1063.

Wang, N., & Li, Q. (2022). Study on extraction and antioxidant activity of polysaccharides from Radix Bupleuri by natural deep eutectic solvents combined with ultrasound-assisted enzymolysis. *Sustainable Chemistry and Pharmacy*, *30*, 100877. <https://doi.org/https://doi.org/10.1016/j.scp.2022.100877>

Wen, Y., & Chen, G. (2025). Optimization of ultrasound-assisted deep eutectic solvent extraction, characterization, and bioactivities of polysaccharide from the fruit of Rosa roxburghii Tratt. *Journal of Food Measurement and Characterization*, 1–15.

Xiong, B., Zhang, W., Wu, Z., Liu, R., Yang, C., Hui, A., Huang, X., & Xian, Z. (2021). Preparation, characterization, antioxidant and anti-inflammatory activities of acid-soluble pectin from okra (Abelmoschus esculentus L.). *International Journal of Biological Macromolecules*, *181*, 824–834.

Xue, J., Su, J., Wang, X., Zhang, R., Li, X., Li, Y., Ding, Y., & Chu, X. (2024). Eco-friendly and efficient extraction of polysaccharides from Acanthopanax senticosus by ultrasound-assisted deep eutectic solvent. *Molecules*, *29*(5), 942.

Yuan, Q., Lin, S., Fu, Y., Nie, X.-R., Liu, W., Su, Y., Han, Q.-H., Zhao, L., Zhang, Q., & Lin, D.-R. (2019). Effects of extraction methods on the physicochemical characteristics and biological activities of polysaccharides from okra (Abelmoschus esculentus). *International Journal of Biological Macromolecules*, *127*, 178–186.

Zhang, H., Lin, Y., Bao, Y., Li, W.-J., Hong, B., & Zhao, M. (2024). A high selective separation method for high-purity polysaccharides from dandelions by density-oriented deep eutectic solvent ultrasonic-assisted system. *Sustainable Chemistry and Pharmacy*, *42*, 101844. <https://doi.org/https://doi.org/10.1016/j.scp.2024.101844>

Zhang, Y., He, L., Li, Q., Cheng, J., Wang, Y., Zhao, J., Yuan, S., Chen, Y., & Shi, R. (2022). Optimization of ultrasonic-assisted deep eutectic solvent for the extraction of polysaccharides from Indocalamus tessellatus leaves and their biological studies. *Sustainable Chemistry and Pharmacy*, *30*, 100855. <https://doi.org/https://doi.org/10.1016/j.scp.2022.100855>

Zhou, B., Jiang, J., Huo, Y., Lin, J., & Shang, C. (2025). Extraction, purification and antioxidant activity of Chlorella polysaccharide. *Algal Research*, *89*, 104077. <https://doi.org/https://doi.org/10.1016/j.algal.2025.104077>
